# Supplementary material for: Malaria treatment-seeking behaviour and its associated factors: A cross-sectional study in rural East Nusa Tenggara Province, Indonesia
Source: PLoS One. 2022 Feb 4;17(2):e0263178. doi: 10.1371/journal.pone.0263178 (PMC8815915; doi:10.1371/journal.pone.0263178)
Supplement: S1 Dataset — (PDF) [file pone.0263178.s002.pdf]

RID	G	AG	D	Edu	Eth	Job	SES	FZ	HF	DHF	Inc1	Inc2	Q1	Q2	Q3	Q4	O1	O2	O3
10111	1	4	2	3	3	2	3	2	1	2	1	1	1	1	1	1	0	0	0
10112	1	2	2	5	3	5	3	1	1	2	0	0	1	1	1	1	0	0	0
10113	1	3	2	2	3	2	2	1	1	2	0	0	1	1	1	1	0	0	0
10114	1	1	2	5	3	5	2	1	1	2	0	0	1	1	1	1	0	0	0
10115	1	4	2	3	3	5	2	1	1	2	1	1	1	1	1	1	0	0	0
10116	1	3	2	3	3	2	2	1	1	2	0	0	1	1	1	1	0	0	0
10117	1	1	2	5	3	2	2	1	1	2	0	0	1	1	1	1	0	0	0
10118	1	3	2	4	3	5	2	1	1	2	1	0	1	1	1	1	0	0	0
10119	1	3	2	3	3	2	2	1	1	2	0	0	1	1	1	1	0	0	0
101110	1	4	2	2	3	2	2	1	1	2	0	0	1	1	1	1	0	0	0
101111	1	3	2	2	3	2	2	2	1	2	0	0	1	1	1	1	0	0	0
101112	1	1	2	5	3	5	2	1	1	2	1	0	1	1	1	1	0	0	0
101113	1	4	2	4	3	5	2	1	2	2	1	1	1	1	1	1	0	0	0
101114	1	5	2	5	3	5	3	1	1	2	1	1	1	1	1	1	0	0	0
101115	0	5	2	1	3	2	1	1	1	2	0	0	0	99	99	99	99	99	99
101116	0	2	2	3	3	1	2	2	1	1	0	0	1	1	1	1	0	0	0
101117	1	3	2	5	3	5	3	1	1	1	1	1	1	1	2	1	1	0	1
101118	1	4	2	3	3	2	2	1	1	1	0	0	1	1	1	1	0	0	0
101119	1	2	2	3	3	2	2	2	1	1	0	0	1	1	1	1	0	0	0
101120	0	4	2	2	3	1	1	1	1	1	0	0	1	1	1	1	0	0	0
101121	1	3	2	3	3	2	1	1	1	1	0	0	1	1	1	1	0	0	0
101122	0	4	2	1	3	1	1	1	1	1	0	0	1	1	1	1	0	0	0
101123	0	4	2	3	3	1	2	1	1	1	0	0	1	1	1	1	0	0	0
101124	0	4	2	1	3	1	2	1	1	1	0	0	1	1	1	1	0	0	0
101125	1	5	2	1	3	2	2	1	1	1	0	0	1	1	1	1	0	0	0
101126	0	3	2	3	3	1	2	1	1	1	0	0	1	1	1	1	0	0	0
101127	1	3	2	3	3	2	2	1	1	1	0	0	1	1	1	1	0	0	0
101128	1	4	2	3	3	2	2	1	1	1	0	0	1	1	1	1	0	0	0
101129	0	2	2	4	3	1	1	1	1	1	0	0	1	1	1	1	0	0	0
101130	1	4	2	3	3	2	2	1	1	1	0	0	1	1	1	1	0	0	0
10121	0	1	2	3	3	1	2	1	3	3	0	0	1	1	1	1	0	0	0
10122	1	2	2	4	3	2	2	2	3	3	0	0	1	1	1	1	0	0	0
10123	0	2	2	2	3	1	2	2	3	3	0	0	1	1	1	1	0	0	0
10124	0	1	2	4	3	1	2	1	3	3	0	0	1	1	1	1	0	0	0
10125	0	1	2	4	3	1	2	1	3	3	0	0	1	1	1	1	0	0	0
10126	0	5	2	1	3	2	1	1	3	3	0	0	0	99	99	99	99	99	99
10127	1	5	2	1	3	2	1	1	3	3	0	0	1	1	3	1	1	0	1
10128	0	3	2	2	3	1	2	1	3	2	0	0	1	1	1	1	0	0	0
10129	1	3	2	1	3	2	2	1	3	2	0	0	1	1	3	1	1	0	1
101210	0	2	2	1	3	1	2	2	3	2	0	0	1	1	1	1	0	0	0
101211	0	4	2	1	3	1	2	1	3	2	0	0	1	1	1	1	0	0	0
101212	0	2	2	4	3	1	3	1	3	2	0	0	1	1	3	1	1	0	1
101213	1	5	2	2	3	2	2	1	3	2	0	0	1	1	3	1	1	0	1
101214	1	4	2	1	3	2	2	1	3	2	0	0	1	1	1	1	0	0	0
101215	0	1	2	1	3	1	2	1	3	2	0	0	0	99	99	99	99	99	99
101216	0	5	2	1	3	1	1	1	3	2	0	0	1	1	1	1	0	0	0
101217	1	2	2	2	3	5	2	1	3	2	0	0	1	1	1	1	0	0	0
101218	0	1	2	2	3	1	2	1	3	2	0	0	1	1	1	1	0	0	0
101219	1	2	2	3	3	4	2	1	3	3	0	0	1	1	1	1	0	0	0
101220	0	2	2	2	3	1	2	1	3	3	0	0	1	1	1	1	0	0	0
101221	1	2	2	3	3	4	2	1	3	3	0	0	1	1	1	1	0	0	0
101222	1	2	2	4	3	4	3	1	3	3	1	0	1	1	1	1	0	0	0
101223	1	4	2	1	3	4	2	1	3	3	0	0	1	1	1	1	0	0	0
101224	0	3	2	2	3	1	2	1	3	2	0	0	1	1	1	1	0	0	0
101225	1	1	2	3	3	4	2	1	3	3	0	0	1	1	1	1	0	0	0

101226	0	3	2	2	2	1	1	1	4	1	0	0	1	1	3	1	1	0	1
101227	1	2	2	3	2	4	2	1	3	1	0	0	1	1	1	1	0	0	0
101228	0	3	2	3	3	1	2	1	3	1	0	0	1	1	1	1	0	0	0
101229	1	2	2	4	3	1	2	1	3	1	1	0	1	1	1	1	0	0	0
101230	1	3	2	3	3	2	3	1	3	1	0	0	1	1	1	1	0	0	0
101231	1	5	2	1	3	2	1	1	3	1	0	0	0	99	99	99	99	99	99
101232	1	5	2	1	3	2	1	1	3	2	0	0	1	1	1	1	0	0	0
101233	1	2	2	1	3	2	1	1	3	1	0	0	1	1	1	1	0	0	0
101234	1	3	2	2	3	2	1	2	3	3	0	0	1	1	1	1	0	0	0
101235	1	5	2	1	3	2	1	1	3	3	0	0	1	1	1	1	0	0	0
10131	0	3	2	3	3	1	2	1	4	1	0	0	1	1	1	1	0	0	0
10132	1	1	2	4	3	5	2	1	4	1	0	0	1	1	1	1	0	0	0
10133	1	3	2	4	3	5	2	1	4	1	0	0	1	1	1	1	0	0	0
10134	0	2	2	4	3	1	2	1	4	1	1	0	1	1	1	1	0	0	0
10135	0	1	2	4	3	1	2	1	4	1	0	0	1	1	1	1	0	0	0
10136	0	3	2	4	3	1	2	1	4	1	0	0	1	1	1	1	0	0	0
10137	1	2	2	4	3	5	3	2	4	2	0	0	1	1	1	1	0	0	0
10138	0	2	2	4	3	1	2	1	4	2	1	0	1	1	1	1	0	0	0
10139	0	2	2	3	3	1	2	1	4	2	1	0	1	1	1	1	0	0	0
101310	1	2	2	4	3	5	2	1	4	2	0	0	1	1	1	1	0	0	0
101311	1	1	2	4	3	5	2	1	4	2	0	0	1	1	1	1	0	0	0
101312	0	2	2	4	3	1	2	1	4	2	0	0	1	1	1	1	0	0	0
101313	0	3	2	1	3	1	1	1	4	2	0	0	1	1	1	1	0	0	0
101314	1	2	2	4	3	2	2	1	4	2	0	0	1	1	1	1	0	0	0
101315	1	1	2	3	3	2	1	1	4	2	0	0	1	1	1	1	0	0	0
101316	0	3	2	3	3	1	2	1	4	2	0	0	1	1	1	1	0	0	0
101317	0	2	2	3	3	1	2	1	4	2	0	0	1	1	1	1	0	0	0
101318	0	4	2	4	3	1	2	2	4	2	0	0	1	1	1	1	0	0	0
101319	1	3	2	3	3	3	3	1	4	2	0	0	1	1	1	1	0	0	0
101320	1	5	2	2	3	2	2	1	4	2	0	0	1	1	1	1	0	0	0
101321	0	4	2	2	3	1	2	1	4	2	0	0	1	1	1	1	0	0	0
101322	1	2	2	3	3	2	2	1	4	2	0	0	1	1	1	1	0	0	0
101323	0	2	2	1	3	2	2	2	4	2	0	0	1	1	1	1	0	0	0
101324	1	4	2	2	3	2	2	1	4	2	0	0	1	1	1	1	0	0	0
101325	1	3	2	3	3	1	2	1	4	2	0	0	1	1	1	1	0	0	0
101326	0	1	2	1	3	1	2	1	4	2	0	0	1	1	1	1	0	0	0
101327	1	1	2	3	3	2	2	1	4	2	0	0	1	1	1	1	0	0	0
101328	1	5	2	1	3	2	2	1	4	2	0	0	1	1	1	1	0	0	0
101329	0	1	2	4	3	1	2	1	4	2	0	0	1	1	1	1	0	0	0
101330	1	5	2	3	3	2	2	1	4	2	0	0	1	1	1	1	0	0	0
10141	1	2	2	5	3	5	3	1	4	1	0	0	1	1	1	1	0	0	0
10142	0	2	2	4	3	1	2	1	4	1	0	0	1	1	3	1	1	0	1
10143	1	5	2	2	3	5	2	1	4	1	1	1	1	1	3	2	1	0	1
10144	0	1	2	4	3	5	2	1	4	1	0	0	1	1	3	1	1	0	1
10145	1	4	2	1	3	2	1	2	4	1	0	0	0	99	99	99	99	99	99
10146	0	5	2	2	2	1	1	2	4	1	0	0	0	99	99	99	99	99	99
10147	1	2	2	4	3	5	2	2	4	1	0	0	1	1	2	1	1	0	1
10148	1	4	2	1	3	2	2	2	4	1	0	0	1	1	3	6	1	1	1
10149	0	3	2	2	3	1	3	2	4	2	0	0	1	1	1	1	0	0	0
101410	0	3	2	2	2	1	2	2	4	2	0	0	1	0	5	5	1	1	1
101411	1	5	2	1	3	5	1	1	4	2	0	0	0	99	99	99	99	99	99
101412	1	3	2	2	3	2	2	1	4	2	0	0	1	1	5	5	1	1	1
101413	1	2	2	4	3	2	2	1	4	2	0	0	1	1	1	1	0	0	0
101414	1	3	2	3	3	2	1	1	4	2	0	0	0	99	99	99	99	99	99
101415	1	4	2	1	3	2	1	1	4	2	0	0	0	99	99	99	99	99	99
101416	0	4	2	1	3	1	1	1	4	2	0	0	0	99	99	99	99	99	99

101417	1	3	2	2	3	2	2	1	4	2	0	0	1	1	2	1	1	0	1
101418	1	4	2	2	3	2	1	1	4	2	0	0	1	1	3	1	1	0	1
101419	1	3	2	2	3	2	2	1	4	2	0	0	1	1	2	2	1	0	1
101420	0	2	2	3	3	1	2	2	4	2	0	0	1	1	3	1	1	0	1
101421	1	4	2	2	3	2	2	1	4	2	0	0	0	99	99	99	99	99	99
101422	0	3	2	1	3	1	1	1	4	2	0	0	0	99	99	99	99	99	99
101423	1	4	2	2	3	2	2	1	4	2	0	0	1	1	2	1	1	0	1
101424	0	1	2	4	2	1	2	1	4	1	0	0	1	1	2	1	1	0	1
101425	1	1	2	4	3	4	2	1	4	1	0	0	1	1	3	1	1	0	1
101426	0	1	2	3	3	1	1	1	4	2	0	0	1	1	1	1	0	0	0
101427	0	3	2	3	3	1	2	1	4	2	0	0	1	1	2	1	1	0	1
101428	0	4	2	1	3	1	2	2	4	2	0	0	1	1	2	1	1	0	1
101429	1	2	2	3	3	2	2	1	4	2	0	0	1	1	3	1	1	0	1
101430	0	3	2	2	3	1	2	1	4	2	0	0	1	1	2	1	1	0	1
101431	1	4	2	2	3	2	2	2	4	2	0	0	1	1	2	1	1	0	1
101432	1	4	2	1	3	1	2	1	4	2	0	0	1	1	3	1	1	0	1
101433	0	1	2	3	3	1	2	1	4	2	0	0	1	1	2	1	1	0	1
101434	1	2	2	3	4	2	1	1	4	2	0	0	1	1	1	1	0	0	0
101435	1	2	2	3	3	4	2	1	4	2	0	0	1	1	2	1	1	0	1
10151	0	2	2	4	3	1	2	2	4	2	0	0	1	1	2	1	1	0	1
10152	1	4	2	4	3	4	2	1	4	2	0	0	1	1	3	2	1	0	1
10153	0	2	2	3	3	1	2	1	4	2	0	0	1	1	2	1	1	0	1
10154	0	1	2	4	3	5	2	2	4	2	1	0	1	1	2	2	1	0	1
10155	1	3	2	3	3	4	2	1	4	2	0	0	1	1	1	1	0	0	0
10156	1	3	2	3	3	5	2	1	4	2	0	0	1	1	2	1	1	0	1
10157	0	1	2	4	3	1	2	1	1	1	0	0	1	1	2	1	1	0	1
10158	0	2	2	4	3	1	2	1	1	1	0	0	1	1	3	1	1	0	1
10159	1	5	2	4	3	5	3	1	4	3	1	1	1	1	2	1	1	0	1
101510	1	1	2	4	3	4	2	1	1	1	0	0	1	1	2	1	1	0	1
101511	0	4	2	4	3	5	3	2	1	1	1	1	1	1	3	1	1	0	1
101512	0	2	2	5	3	1	1	1	1	1	0	0	1	1	2	4	1	1	1
101513	1	3	2	4	3	4	2	2	1	2	1	0	1	1	2	1	1	0	1
101514	1	5	2	3	3	4	2	1	1	2	0	0	1	1	1	1	0	0	0
101515	0	1	2	4	3	1	2	1	1	1	0	0	1	1	2	1	1	0	1
101516	1	2	2	2	3	4	2	1	1	1	0	0	1	1	2	2	1	0	1
101517	0	2	2	3	2	1	2	2	1	1	0	0	1	1	3	1	1	0	1
101518	1	3	2	4	3	4	2	1	1	1	0	0	1	1	2	1	1	0	1
101519	0	2	2	4	3	1	2	2	1	1	0	0	1	1	2	1	1	0	1
101520	1	4	2	4	3	4	3	2	1	2	1	1	1	1	2	1	1	0	1
101521	0	5	2	2	3	1	2	1	1	2	0	0	1	1	3	1	1	0	1
101522	1	5	2	3	3	5	2	1	1	2	1	1	1	1	2	1	1	0	1
101523	1	4	2	4	3	5	2	1	1	1	1	1	1	1	2	2	1	0	1
101524	0	3	2	4	3	5	3	1	1	1	1	1	1	1	3	2	1	0	1
101525	1	2	2	4	3	5	2	2	1	1	1	1	1	1	2	1	1	0	1
101526	1	2	2	4	3	5	2	1	1	1	1	1	1	1	2	1	1	0	1
101527	1	3	2	4	3	5	2	1	1	1	0	0	1	1	2	1	1	0	1
101528	1	4	2	2	3	2	2	1	1	1	1	1	1	1	2	1	1	0	1
101529	0	3	2	3	3	1	2	1	1	1	0	0	1	1	3	1	1	0	1
101530	0	3	2	4	3	1	2	1	1	1	0	0	1	1	2	1	1	0	1
10161	0	1	2	4	3	1	2	1	1	1	0	0	1	1	2	1	1	0	1
10162	0	1	2	2	3	1	2	1	2	2	1	0	1	1	2	1	1	0	1
10163	0	2	2	4	3	1	3	1	1	2	0	0	1	1	3	1	1	0	1
10164	1	4	2	5	3	5	3	2	1	2	1	1	1	1	1	6	0	1	1
10165	1	2	2	2	3	2	2	1	1	2	0	0	1	1	2	1	1	0	1
10166	1	5	2	2	3	2	2	1	1	2	1	1	1	1	2	1	1	0	1
10167	1	4	2	2	3	4	2	2	1	1	0	0	1	1	2	1	1	0	1

10168	1	1	2	2	3	2	2	1	1	1	0	0	1	1	3	1	1	0	1
10169	0	3	2	2	3	1	2	1	1	1	0	0	1	1	3	2	1	0	1
101610	0	3	2	1	3	1	2	2	1	1	0	0	1	1	2	1	1	0	1
101611	0	1	2	2	3	1	2	2	1	1	0	0	1	1	2	6	1	1	1
101612	0	4	2	2	3	1	2	1	1	1	0	0	1	1	4	6	1	1	1
101613	1	3	2	2	3	4	2	1	1	1	0	0	1	1	2	1	1	0	1
101614	0	3	2	2	3	1	3	2	1	1	0	0	1	1	2	1	1	0	1
101615	0	4	2	2	3	1	2	1	1	1	0	0	1	1	3	1	1	0	1
101616	0	1	2	4	3	1	2	1	1	1	0	0	1	1	2	1	1	0	1
101617	0	3	2	1	3	1	2	2	1	1	0	0	1	1	2	1	1	0	1
101618	1	5	2	2	3	2	1	1	1	1	0	0	1	1	3	2	1	0	1
101619	1	4	2	2	3	4	2	2	1	1	0	0	1	1	3	6	1	1	1
101620	0	1	2	3	3	1	2	2	1	1	0	0	1	1	3	1	1	0	1
101621	1	4	2	2	3	4	2	1	1	1	0	0	1	1	2	1	1	0	1
101622	0	2	2	3	3	1	2	1	1	1	0	0	1	1	2	1	1	0	1
101623	1	1	2	2	3	4	2	1	1	1	0	0	1	1	3	1	1	0	1
101624	0	5	2	2	3	1	1	1	1	1	0	0	1	1	2	6	1	1	1
101625	1	2	2	3	1	2	2	1	1	1	0	0	1	1	3	1	1	0	1
101626	1	4	2	2	3	4	2	2	1	1	0	0	1	1	2	1	1	0	1
101627	1	3	2	2	3	4	2	1	1	1	0	0	1	1	2	1	1	0	1
101628	1	2	2	2	3	4	2	2	1	1	0	0	1	1	2	6	1	1	1
101629	1	3	2	3	3	4	2	1	1	1	0	0	1	1	2	1	1	0	1
101630	0	4	2	2	3	1	2	1	1	1	0	0	1	1	2	1	1	0	1
10271	0	3	2	1	3	1	2	2	1	1	0	0	1	1	3	4	1	1	1
10272	0	1	2	4	3	1	2	2	1	1	0	0	1	1	2	1	1	0	1
10273	0	2	2	2	3	1	2	2	1	3	0	0	1	1	3	1	1	0	1
10274	1	2	2	2	3	4	2	2	1	3	0	0	1	1	2	1	1	0	1
10275	0	4	2	2	3	1	1	1	1	3	0	0	1	1	1	5	0	1	1
10276	1	2	2	1	3	2	2	1	1	3	0	0	1	0	5	5	1	1	1
10277	0	1	2	3	3	1	2	2	1	3	0	0	1	1	3	6	1	1	1
10278	0	5	2	1	3	1	1	1	1	1	0	0	0	99	99	99	99	99	99
10279	1	4	2	2	3	2	2	2	1	3	0	0	1	1	1	1	0	0	0
102710	1	3	2	4	3	2	2	1	1	3	0	0	1	1	2	1	1	0	1
102711	1	4	2	1	3	2	2	1	1	3	0	0	1	1	4	4	1	1	1
102712	0	2	2	4	3	1	2	1	1	3	0	0	1	1	1	1	0	0	0
102713	0	4	2	2	3	1	2	1	1	3	0	0	0	99	99	99	99	99	99
102714	0	3	2	3	3	1	1	1	1	3	0	0	1	1	3	4	1	1	1
102715	1	5	2	1	3	2	1	1	1	3	0	0	0	99	99	99	99	99	99
102716	1	4	2	2	3	2	1	1	1	3	0	0	1	1	3	6	1	1	1
102717	0	4	2	2	3	1	1	1	1	3	0	0	0	99	99	99	99	99	99
102718	1	3	2	2	3	2	1	1	1	3	0	0	1	1	1	1	0	0	0
102719	0	4	2	1	3	2	1	1	1	3	0	0	1	1	2	5	1	1	1
102720	1	3	2	3	3	4	2	1	1	3	0	0	1	1	2	6	1	1	1
102721	1	4	2	2	3	2	2	1	1	3	0	0	1	1	1	1	0	0	0
102722	0	4	2	1	3	1	1	1	1	3	0	0	0	99	99	99	99	99	99
102723	0	2	2	2	3	1	1	1	1	3	0	0	1	1	3	5	1	1	1
102724	1	3	2	1	3	2	2	1	1	3	0	0	1	1	2	1	1	0	1
102725	0	2	2	2	3	1	1	2	1	3	0	0	1	1	4	1	1	0	1
102726	1	3	2	2	3	2	2	1	1	3	0	0	1	1	3	7	1	1	1
102727	1	2	2	2	3	2	2	1	1	2	0	0	1	1	1	1	0	0	0
102728	1	2	2	4	3	2	2	1	1	2	0	0	1	1	1	1	0	0	0
102729	1	2	2	3	3	4	2	2	1	3	0	0	1	1	2	6	1	1	1
102730	1	3	2	2	3	4	2	1	1	3	0	0	1	1	2	6	1	1	1
10281	1	3	2	2	3	2	2	1	1	4	0	0	1	1	2	6	1	1	1
10282	1	4	2	2	3	2	1	1	1	1	0	0	0	99	99	99	99	99	99
10283	0	4	2	1	3	1	1	1	1	4	0	0	1	1	3	5	1	1	1

10284	1	3	2	1	3	1	2	1	1	4	0	0	1	1	2	5	1	1	1
10285	1	4	2	2	3	2	2	1	1	4	0	0	1	1	1	4	0	1	1
10286	0	3	2	2	3	1	1	1	1	4	0	0	1	1	2	4	1	1	1
10287	0	5	2	1	3	1	1	1	1	4	0	0	0	99	99	99	99	99	99
10288	1	5	2	1	3	2	1	1	1	4	0	0	0	99	99	99	99	99	99
10289	1	1	2	3	3	2	1	1	1	4	0	0	1	1	3	1	1	0	1
102810	0	3	2	2	3	1	1	1	1	4	0	0	1	1	2	1	1	0	1
102811	1	3	2	3	3	2	2	1	1	4	0	0	1	1	1	2	0	0	0
102812	1	3	2	3	3	2	2	1	1	4	0	0	1	1	1	1	0	0	0
102813	1	4	2	2	3	2	1	1	1	4	0	0	1	1	5	4	1	1	1
102814	0	3	2	2	3	1	1	1	1	4	0	0	0	99	99	99	99	99	99
102815	1	5	2	1	3	2	1	1	1	4	0	0	0	99	99	99	99	99	99
102816	1	3	2	2	3	2	1	1	1	4	0	0	1	1	1	1	0	0	0
102817	0	4	2	2	3	1	1	1	1	4	0	0	0	99	99	99	99	99	99
102818	0	4	2	2	3	1	1	1	1	4	0	0	1	1	2	1	1	0	1
102819	1	3	2	2	3	2	1	1	1	4	0	0	1	1	3	6	1	1	1
102820	0	2	2	3	3	1	1	1	1	4	0	0	1	1	3	1	1	0	1
102821	1	2	2	3	3	2	3	1	1	1	0	0	1	1	2	1	1	0	1
102822	0	3	2	2	3	1	2	2	1	1	0	0	1	1	2	5	1	1	1
102823	0	3	2	2	3	1	3	1	1	1	0	0	1	1	1	1	0	0	0
102824	1	4	2	1	3	2	2	1	1	1	0	0	0	99	99	99	99	99	99
102825	1	4	2	2	3	2	2	1	1	1	0	0	1	1	1	1	0	0	0
102826	0	4	2	2	3	1	2	1	1	1	0	0	1	1	1	1	0	0	0
102827	0	5	2	2	3	1	3	1	1	1	0	0	1	1	1	1	0	0	0
102828	0	2	2	2	3	1	3	1	1	1	0	0	1	1	1	1	0	0	0
102829	1	5	2	1	3	2	3	1	1	1	0	0	1	1	1	1	0	0	0
102830	0	1	2	3	3	1	2	1	1	1	0	0	1	1	1	1	0	0	0
10291	1	5	2	2	3	2	2	1	1	2	0	0	1	1	1	6	0	1	1
10292	0	4	2	2	3	1	2	1	1	2	0	0	0	99	99	99	99	99	99
10293	0	5	2	1	3	1	2	1	1	2	0	0	0	99	99	99	99	99	99
10294	1	1	2	3	3	2	2	1	1	2	0	0	1	1	1	1	0	0	0
10295	1	2	2	3	3	2	2	1	1	2	0	0	1	1	1	5	0	1	1
10296	1	5	2	2	3	2	3	2	1	2	0	0	1	1	3	1	1	0	1
10297	1	3	2	2	3	2	2	1	1	2	0	0	0	99	99	99	99	99	99
10298	0	1	2	4	3	1	3	1	1	2	0	0	1	1	2	1	1	0	1
10299	1	4	2	2	3	2	2	1	1	2	0	0	1	1	3	5	1	1	1
102910	0	3	2	2	3	1	2	2	1	2	0	0	1	1	1	1	0	0	0
102911	1	3	2	3	3	2	2	1	1	2	0	0	1	1	3	1	1	0	1
102912	0	1	2	3	3	1	2	1	1	2	0	0	1	1	2	5	1	1	1
102913	1	3	2	2	3	2	2	1	1	2	0	0	1	1	3	1	1	0	1
102914	0	4	2	2	3	1	2	1	1	2	0	0	1	1	1	1	0	0	0
102915	0	4	2	1	3	1	2	1	1	2	0	0	1	1	2	1	1	0	1
102916	1	4	2	2	3	2	2	1	1	2	0	0	0	99	99	99	99	99	99
102917	1	3	2	2	3	2	2	1	1	2	0	0	1	1	1	5	0	1	1
102918	0	4	2	2	3	1	2	1	1	2	0	0	0	99	99	99	99	99	99
102919	0	4	2	2	3	1	2	1	1	2	0	0	0	99	99	99	99	99	99
102920	0	1	2	3	3	1	2	1	1	2	0	0	1	1	3	1	1	0	1
102921	0	1	2	2	3	1	2	1	1	2	0	0	1	1	2	1	1	0	1
102922	1	3	2	2	3	2	2	1	1	2	0	0	1	1	3	5	1	1	1
102923	1	3	2	2	3	2	2	1	1	2	0	0	1	1	1	1	0	0	0
102924	1	2	2	3	3	2	2	1	1	2	0	0	0	99	99	99	99	99	99
102925	0	2	2	4	3	1	3	1	1	2	0	0	1	1	2	1	1	0	1
102926	1	3	2	2	3	2	2	1	1	2	0	0	1	1	3	1	1	0	1
102927	1	3	2	2	3	2	2	1	1	2	0	0	0	99	99	99	99	99	99
102928	0	4	2	3	3	1	2	1	1	2	0	0	1	1	2	5	1	1	1
102929	0	4	2	2	3	1	2	1	1	2	0	0	1	1	2	6	1	1	1

102930	1	4	2	2	3	2	2	1	1	2	0	0	1	1	2	5	1	1	1
102101	0	1	2	4	3	1	2	2	1	1	0	0	1	1	1	1	0	0	0
102102	0	2	2	3	3	1	2	1	1	1	0	0	1	1	3	1	1	0	1
102103	0	2	2	2	3	1	2	2	2	1	0	0	1	1	3	1	1	0	1
102104	0	5	2	1	3	2	2	2	1	1	0	0	1	1	3	1	1	0	1
102105	1	5	2	3	3	2	3	2	1	1	0	0	1	1	1	1	0	0	0
102106	1	4	2	1	3	2	2	2	4	2	0	0	1	1	3	1	1	0	1
102107	0	1	2	4	3	1	2	1	4	1	0	0	1	1	3	1	1	0	1
102108	0	2	2	2	3	1	2	2	1	1	0	0	1	1	3	1	1	0	1
102109	1	3	2	2	3	2	3	1	1	1	0	0	1	1	3	1	1	0	1
1021010	0	3	2	2	3	1	2	1	1	1	0	0	1	1	3	1	1	0	1
1021011	1	4	2	1	2	2	2	1	1	1	0	0	1	1	3	1	1	0	1
1021012	0	1	2	2	3	1	3	1	1	1	0	0	1	1	3	1	1	0	1
1021013	0	5	2	1	3	2	2	1	4	1	0	0	1	1	4	5	1	1	1
1021014	0	2	2	2	3	1	2	1	1	1	0	0	1	1	3	1	1	0	1
1021015	1	5	2	1	3	2	2	1	1	1	0	0	1	1	1	1	0	0	0
1021016	1	4	2	2	3	2	2	2	4	1	0	0	1	1	3	1	1	0	1
1021017	1	2	2	3	3	4	2	2	2	4	0	0	1	1	3	1	1	0	1
1021018	1	2	2	1	3	2	2	2	1	1	0	0	1	1	3	1	1	0	1
1021019	1	4	2	1	3	2	2	1	1	1	0	0	1	1	3	1	1	0	1
1021020	1	5	2	2	2	2	2	1	1	1	0	0	1	0	5	5	1	1	1
1021021	1	4	2	1	3	2	2	1	1	1	0	0	1	1	3	2	1	0	1
1021022	1	3	2	1	3	2	2	1	1	1	0	0	0	99	99	99	99	99	99
1021023	1	4	2	2	3	5	3	2	4	1	1	0	1	1	4	1	1	0	1
1021024	1	3	2	1	3	2	2	1	1	1	0	0	1	1	3	1	1	0	1
1021025	1	4	2	2	3	5	2	2	4	2	0	0	1	1	3	1	1	0	1
1021026	1	4	2	2	3	2	2	1	1	1	0	0	1	1	3	1	1	0	1
1021027	0	5	2	1	3	2	1	1	1	1	0	0	1	0	5	5	1	1	1
1021028	0	5	2	1	3	2	2	2	4	2	0	0	1	1	1	1	0	0	0
1021029	0	5	2	1	3	2	2	1	1	1	0	0	1	1	1	1	0	0	0
1021030	0	5	2	2	3	2	3	2	1	1	0	0	1	1	1	1	0	0	0
1021031	0	2	2	3	3	1	2	1	2	4	0	0	1	1	3	1	1	0	1
1021032	0	2	2	4	3	1	3	2	4	1	0	0	1	1	3	1	1	0	1
1021033	0	2	2	3	3	1	2	1	1	1	0	0	1	1	1	1	0	0	0
102111	0	3	2	2	3	2	1	1	2	1	0	0	0	99	99	99	99	99	99
102112	1	3	2	2	3	3	2	1	1	1	0	0	1	1	3	1	1	0	1
102113	0	4	2	1	3	2	2	1	2	1	0	0	1	1	2	1	1	0	1
102114	0	3	2	3	3	1	2	1	4	3	0	0	1	1	4	1	1	0	1
102115	1	2	2	2	3	4	2	1	4	3	0	0	1	1	1	1	0	0	0
102116	0	2	2	2	2	1	2	2	4	3	0	0	1	1	3	1	1	0	1
102117	1	2	2	5	3	5	3	1	4	1	1	0	1	1	1	1	0	0	0
102118	1	1	2	3	3	5	2	2	4	4	0	0	1	1	2	5	1	1	1
102119	1	5	2	4	3	4	2	1	2	1	0	0	1	1	1	1	0	0	0
1021110	0	4	2	1	3	2	2	1	4	2	0	0	0	99	99	99	99	99	99
1021111	1	2	2	4	3	4	2	1	4	2	0	0	1	0	5	5	1	1	1
1021112	1	2	2	3	3	2	2	1	4	2	0	0	1	1	1	1	0	0	0
1021113	1	1	2	1	2	4	2	1	2	1	0	0	1	0	5	5	1	1	1
1021114	0	3	2	1	3	2	1	1	2	1	0	0	1	0	5	5	1	1	1
1021115	0	3	2	2	3	1	2	1	2	1	0	0	1	1	3	1	1	0	1
1021116	0	3	2	2	3	1	2	2	1	1	0	0	1	0	5	5	1	1	1
1021117	1	5	2	2	2	5	2	1	4	3	1	1	1	1	1	1	0	0	0
1021118	0	4	2	2	3	1	2	1	4	3	0	0	1	1	1	1	0	0	0
1021119	0	4	2	2	3	1	2	2	4	3	0	0	1	1	1	1	0	0	0
1021120	1	3	2	1	3	2	2	2	4	2	0	0	1	1	4	1	1	0	1
1021121	1	5	2	1	3	2	1	1	4	1	0	0	0	99	99	99	99	99	99
1021122	1	5	2	1	3	2	3	2	4	2	0	0	1	1	3	1	1	0	1

1021123	0	5	2	2	3	2	1	1	4	3	0	0	1	0	5	5	1	1	1
1021124	1	4	2	1	3	4	2	2	4	4	0	0	1	1	2	1	1	0	1
1021125	1	2	2	3	3	4	3	1	4	2	0	0	1	1	3	1	1	0	1
1021126	0	4	2	2	3	5	3	2	4	3	0	0	1	1	3	1	1	0	1
1021127	1	3	2	1	3	4	2	1	4	2	0	0	1	1	1	1	0	0	0
1021128	1	3	2	4	3	5	3	2	4	1	0	0	1	1	1	1	0	0	0
1021129	1	3	2	4	3	5	3	2	4	1	0	0	1	1	3	1	1	0	1
1021130	1	3	2	4	3	5	3	1	4	3	0	0	1	1	1	1	0	0	0
1021131	0	2	2	4	3	5	2	1	4	3	0	0	1	1	1	1	0	0	0
1021132	0	3	2	4	3	1	3	2	4	2	0	0	1	1	3	1	1	0	1
1021133	0	4	2	2	3	5	3	1	4	3	0	0	1	1	1	1	0	0	0
1021134	1	4	2	4	3	5	3	2	4	2	0	0	1	1	1	1	0	0	0
1021135	1	2	2	5	2	5	3	1	4	2	0	0	1	1	1	1	0	0	0
1021136	0	2	2	4	3	1	2	2	4	4	0	0	1	1	1	1	0	0	0
1021137	0	5	2	2	3	2	2	2	4	3	0	0	1	1	1	1	0	0	0
1021138	1	2	2	5	2	5	3	1	4	2	0	0	1	1	1	1	0	0	0
1021139	0	4	2	2	3	1	2	1	4	2	0	0	1	1	1	1	0	0	0
102121	0	5	2	1	3	2	2	1	2	1	0	0	0	99	99	99	99	99	99
102122	0	4	2	2	3	2	3	1	1	1	0	0	1	1	1	1	0	0	0
102123	1	5	2	5	2	5	3	1	2	1	0	0	1	1	1	1	0	0	0
102124	1	4	2	1	3	2	2	1	1	1	0	0	0	99	99	99	99	99	99
102125	0	3	2	3	3	1	2	2	4	1	0	0	1	1	3	1	1	0	1
102126	1	3	2	2	3	2	2	1	2	1	0	0	1	1	3	6	1	1	1
102127	0	3	2	1	3	1	2	2	1	1	0	0	1	1	3	1	1	0	1
102128	1	3	2	3	3	2	3	1	4	1	0	0	1	1	3	1	1	0	1
102129	0	1	2	4	3	1	3	1	4	1	0	0	1	1	3	1	1	0	1
1021210	1	3	2	2	3	2	3	2	2	1	0	0	1	1	1	1	0	0	0
1021211	0	1	2	5	3	5	3	1	2	3	0	0	1	1	1	1	0	0	0
1021212	1	2	2	2	3	2	3	2	2	3	0	0	1	1	2	1	1	0	1
1021213	0	5	2	2	3	1	2	2	2	2	0	0	1	1	1	1	0	0	0
1021214	0	4	2	2	3	1	2	2	2	2	0	0	1	1	4	1	1	0	1
1021215	0	4	2	2	3	1	2	2	2	2	0	0	1	1	4	1	1	0	1
1021216	1	5	2	1	3	2	2	2	2	2	0	0	1	1	3	5	1	1	1
1021217	0	4	2	2	3	1	2	1	2	1	0	0	1	1	4	1	1	0	1
1021218	1	1	2	3	3	4	3	1	2	1	0	0	1	1	3	5	1	1	1
1021219	1	3	2	3	3	2	2	2	2	1	0	0	1	1	1	5	0	1	1
1021220	1	4	2	2	3	2	2	1	2	1	0	0	1	0	5	5	1	1	1
1021221	1	5	2	2	3	2	3	1	1	1	0	0	1	1	1	1	0	0	0
1021222	0	4	2	1	3	1	2	2	2	2	0	0	1	1	1	1	0	0	0
1021223	1	2	2	5	3	5	3	1	4	1	0	0	1	1	2	1	1	0	1
1021224	0	4	2	5	3	5	3	2	4	1	1	1	1	1	2	1	1	0	1
1021225	0	5	2	2	3	1	2	2	2	1	0	0	1	1	2	1	1	0	1
1021226	0	4	2	1	3	1	2	1	2	2	0	0	1	0	5	5	1	1	1
1021227	1	2	2	1	3	4	2	1	2	2	0	0	1	1	1	1	0	0	0
1021228	1	5	2	1	3	2	2	2	2	1	0	0	1	0	5	5	1	1	1
1021229	0	2	2	2	4	1	2	2	4	1	0	0	1	1	3	1	1	0	1
1021230	1	2	2	2	3	2	2	2	2	1	0	0	1	1	4	1	1	0	1
1021231	1	3	2	1	3	4	2	2	2	1	0	0	1	1	3	1	1	0	1
1021232	1	4	2	1	2	4	2	1	2	1	0	0	1	0	5	5	1	1	1
1021233	1	1	2	5	3	3	3	1	2	1	1	1	1	1	2	1	1	0	1
1021234	0	5	2	1	2	1	2	2	4	2	0	0	1	1	1	1	0	0	0
1021235	0	2	2	5	3	5	2	2	4	2	0	0	1	1	1	1	0	0	0
103141	1	4	2	3	3	2	2	1	2	1	1	1	0	99	99	99	99	99	99
103142	1	2	2	3	3	2	2	2	2	1	0	0	0	99	99	99	99	99	99
103143	1	5	2	3	3	2	2	2	2	1	1	0	0	99	99	99	99	99	99
103144	1	3	2	3	3	2	2	2	2	1	0	0	1	1	1	1	0	0	0

103145	1	3	2	3	3	2	2	1	2	1	0	0	0	99	99	99	99	99	99
103146	1	5	2	1	3	2	2	1	2	1	0	0	0	99	99	99	99	99	99
103147	1	5	2	1	3	2	1	2	2	1	0	0	0	99	99	99	99	99	99
103148	1	4	2	3	3	2	2	2	2	1	0	0	0	99	99	99	99	99	99
103149	1	3	2	2	3	2	2	2	2	1	1	0	0	99	99	99	99	99	99
1031410	1	4	2	4	3	2	2	1	2	1	0	0	1	1	1	1	0	0	0
1031411	0	5	2	2	3	1	2	1	3	1	1	1	0	99	99	99	99	99	99
1031412	0	3	2	2	3	1	1	1	3	1	0	0	0	99	99	99	99	99	99
1031413	0	4	2	1	3	1	2	1	3	1	0	0	0	99	99	99	99	99	99
1031414	0	3	2	2	3	1	2	1	3	1	0	0	0	99	99	99	99	99	99
1031415	0	3	2	1	3	1	2	1	3	1	0	0	1	1	1	1	0	0	0
1031416	0	3	2	2	3	1	2	1	3	1	0	0	0	99	99	99	99	99	99
1031417	0	5	2	2	3	1	1	1	3	1	0	0	0	99	99	99	99	99	99
1031418	0	5	2	2	3	1	2	2	3	1	0	0	0	99	99	99	99	99	99
1031419	0	4	2	2	3	1	1	1	3	1	0	0	0	99	99	99	99	99	99
1031420	0	5	2	2	3	1	2	2	3	1	0	0	0	99	99	99	99	99	99
1031421	0	4	2	2	3	1	1	1	3	1	0	0	0	99	99	99	99	99	99
1031422	0	4	2	2	3	1	1	1	3	1	0	0	0	99	99	99	99	99	99
1031423	0	1	2	2	3	1	1	1	3	1	0	0	1	1	1	1	0	0	0
1031424	0	4	2	1	3	1	2	2	3	1	0	0	0	99	99	99	99	99	99
1031425	1	4	2	1	3	2	1	1	3	1	0	0	0	99	99	99	99	99	99
1031426	0	2	2	2	3	1	1	1	3	1	0	0	0	99	99	99	99	99	99
1031427	0	3	2	2	3	1	1	1	3	1	0	0	1	1	1	1	0	0	0
1031428	0	1	2	4	3	1	1	1	3	1	0	0	0	99	99	99	99	99	99
1031429	0	4	2	3	3	1	2	1	3	1	0	0	1	1	3	1	1	0	1
1031430	0	3	2	2	3	1	1	1	3	1	0	0	0	99	99	99	99	99	99
103131	0	3	2	2	3	1	2	1	2	1	0	0	1	1	1	1	0	0	0
103132	0	2	2	5	3	5	2	2	2	4	0	0	1	1	1	1	0	0	0
103133	0	2	2	2	3	1	2	2	2	1	0	0	1	1	4	1	1	0	1
103134	0	2	2	3	3	1	3	2	2	1	0	0	1	1	1	1	0	0	0
103135	0	2	2	5	3	5	3	2	2	1	0	0	1	1	1	1	0	0	0
103136	1	4	2	2	3	2	1	1	2	1	0	0	1	1	4	1	1	0	1
103137	0	3	2	2	3	1	1	2	2	1	0	0	1	1	4	1	1	0	1
103138	0	2	2	2	3	1	1	2	2	1	0	0	0	99	99	99	99	99	99
103139	0	3	2	4	3	1	1	1	2	1	0	0	1	1	1	1	0	0	0
1031310	1	5	2	2	3	2	1	1	2	1	0	0	1	1	3	1	1	0	1
1031311	0	4	2	2	3	1	2	1	2	1	0	0	1	1	3	1	1	0	1
1031312	1	4	2	2	3	5	1	1	2	1	0	0	0	99	99	99	99	99	99
1031313	1	3	2	4	3	2	2	2	2	1	0	0	1	1	4	1	1	0	1
1031314	1	2	2	4	3	2	2	2	2	1	0	0	1	1	4	1	1	0	1
1031315	0	4	2	2	3	1	1	1	2	1	0	0	1	1	3	1	1	0	1
1031316	0	4	2	2	3	1	1	1	2	1	0	0	1	1	4	1	1	0	1
1031317	1	2	2	2	3	2	2	2	2	1	0	0	0	99	99	99	99	99	99
1031318	0	3	2	2	3	2	1	1	2	1	0	0	1	1	4	1	1	0	1
1031319	0	1	2	4	3	5	3	1	2	1	0	0	1	1	4	1	1	0	1
1031320	0	1	2	4	3	1	2	1	2	1	0	0	1	1	1	1	0	0	0
1031321	0	5	2	2	3	1	1	1	2	1	0	0	0	99	99	99	99	99	99
1031322	0	5	2	2	3	1	2	1	2	1	0	0	0	99	99	99	99	99	99
1031323	0	2	2	2	3	1	2	2	2	1	0	0	0	99	99	99	99	99	99
1031324	0	3	2	2	3	1	2	1	2	1	0	0	1	1	4	1	1	0	1
1031325	0	4	2	2	3	1	2	1	2	1	0	0	1	1	3	1	1	0	1
1031326	0	2	2	2	3	1	2	2	2	1	0	0	1	1	1	1	0	0	0
1031327	0	3	2	2	3	1	2	2	2	1	0	0	1	1	4	1	1	0	1
1031328	0	5	2	2	3	1	1	1	2	1	0	0	0	99	99	99	99	99	99
1031329	0	3	2	2	3	1	1	2	2	1	0	0	1	1	3	1	1	0	1
1031330	0	3	2	2	3	1	2	1	2	1	0	0	0	99	99	99	99	99	99

103151	0	4	2	1	3	1	1	1	2	1	0	0	0	99	99	99	99	99	99
103152	0	4	2	2	3	1	2	1	2	1	0	0	1	1	3	1	1	0	1
103153	0	4	2	2	3	1	2	2	2	1	0	0	0	99	99	99	99	99	99
103154	0	1	2	2	3	1	2	2	2	1	0	0	0	99	99	99	99	99	99
103155	0	2	2	2	3	1	2	1	2	1	0	0	1	1	3	1	1	0	1
103156	1	4	2	2	3	2	2	1	2	1	0	0	0	99	99	99	99	99	99
103157	0	1	2	4	3	1	1	1	2	1	0	0	0	99	99	99	99	99	99
103158	0	4	2	2	3	1	1	1	2	1	0	0	0	99	99	99	99	99	99
103159	0	5	2	2	3	1	2	1	2	1	1	1	0	99	99	99	99	99	99
1031510	0	3	2	2	3	1	2	2	2	1	0	0	0	99	99	99	99	99	99
1031511	0	2	2	3	3	1	2	2	2	1	0	0	0	99	99	99	99	99	99
1031512	0	2	2	2	3	1	1	2	2	1	0	0	0	99	99	99	99	99	99
1031513	0	1	2	4	3	1	1	2	2	1	0	0	0	99	99	99	99	99	99
1031514	0	1	2	3	3	1	1	1	2	1	0	0	0	99	99	99	99	99	99
1031515	0	1	2	4	3	1	1	1	2	1	0	0	0	99	99	99	99	99	99
1031516	0	2	2	3	3	1	1	2	2	1	0	0	1	1	2	1	1	0	1
1031517	0	4	2	2	3	1	1	1	2	1	0	0	0	99	99	99	99	99	99
1031518	0	4	2	3	3	1	2	2	2	1	0	0	1	1	1	1	0	0	0
1031519	0	1	2	4	3	1	1	1	2	1	0	0	1	1	1	1	0	0	0
1031520	1	4	2	2	3	2	2	1	2	1	0	0	0	99	99	99	99	99	99
1031521	0	4	2	1	3	1	1	1	2	1	0	0	0	99	99	99	99	99	99
1031522	1	5	2	2	3	2	2	2	2	1	0	0	0	99	99	99	99	99	99
1031523	1	5	2	2	3	2	3	2	2	1	1	0	1	1	1	1	0	0	0
1031524	0	5	2	1	3	1	2	1	2	1	0	0	0	99	99	99	99	99	99
1031525	1	4	2	1	3	2	2	1	2	1	0	0	0	99	99	99	99	99	99
1031526	0	5	2	1	3	1	1	1	2	1	0	0	0	99	99	99	99	99	99
1031527	0	4	2	2	3	1	2	1	2	1	0	0	0	99	99	99	99	99	99
1031528	0	1	2	3	3	1	2	2	2	1	0	0	1	1	1	1	0	0	0
1031529	0	3	2	2	3	1	2	1	2	1	0	0	1	1	2	1	1	0	1
1031530	0	2	2	5	3	5	3	1	2	1	0	0	1	1	1	1	0	0	0
103161	0	3	2	2	3	1	2	2	1	1	0	0	0	99	99	99	99	99	99
103162	0	3	2	3	3	1	2	2	1	1	0	0	0	99	99	99	99	99	99
103163	0	2	2	3	3	1	3	2	1	1	0	0	1	1	1	1	0	0	0
103164	0	2	2	3	3	1	2	1	1	1	0	0	1	1	1	1	0	0	0
103165	0	4	2	4	3	1	1	1	1	1	0	0	1	1	1	1	0	0	0
103166	0	4	2	2	3	1	1	1	1	1	0	0	1	1	1	1	0	0	0
103167	0	2	2	2	3	1	1	2	1	1	0	0	0	99	99	99	99	99	99
103168	0	5	2	2	3	1	2	1	1	1	0	0	1	1	1	1	0	0	0
103169	0	5	2	2	3	1	1	2	1	1	0	0	0	99	99	99	99	99	99
1031610	0	1	2	4	3	1	2	1	1	1	0	0	0	99	99	99	99	99	99
1031611	0	1	2	2	3	1	1	2	1	1	0	0	1	1	4	1	1	0	1
1031612	0	2	2	2	3	1	2	1	1	1	1	1	1	1	3	1	1	0	1
1031613	0	3	2	2	3	1	2	2	1	1	0	0	1	1	4	1	1	0	1
1031614	0	3	2	2	3	1	1	1	1	1	0	0	1	1	2	1	1	0	1
1031615	0	5	2	2	3	1	1	2	1	1	0	0	1	1	1	1	0	0	0
1031616	0	4	2	2	3	1	1	2	1	1	0	0	0	99	99	99	99	99	99
1031617	0	4	2	2	3	1	2	2	1	1	1	1	0	99	99	99	99	99	99
1031618	0	4	2	2	3	1	1	2	1	1	0	0	0	99	99	99	99	99	99
1031619	0	5	2	1	3	1	2	2	1	1	1	1	0	99	99	99	99	99	99
1031620	0	3	2	3	3	1	2	1	1	1	0	0	0	99	99	99	99	99	99
1031621	0	1	2	5	3	5	3	1	1	1	1	1	1	1	2	1	1	0	1
1031622	0	4	2	4	3	1	2	2	1	1	1	1	0	99	99	99	99	99	99
1031623	0	4	2	3	3	1	3	1	1	1	1	1	1	1	1	1	0	0	0
204171	0	1	1	4	1	1	2	2	4	4	0	0	1	1	1	1	0	0	0
204172	0	3	1	1	1	2	2	1	4	4	0	0	1	1	1	1	0	0	0
204173	0	1	1	2	1	1	2	1	4	4	0	0	1	1	2	1	1	0	1

204174	1	2	1	2	1	2	2	2	4	4	0	0	1	1	3	1	1	0	1
204175	0	1	1	4	1	2	2	1	4	4	0	0	1	1	2	1	1	0	1
204176	1	5	1	2	1	2	2	2	4	4	0	0	1	1	2	1	1	0	1
204177	1	4	1	2	1	2	1	2	4	1	0	0	1	1	1	4	0	1	1
204178	1	2	1	1	1	2	2	1	4	4	0	0	1	1	3	1	1	0	1
204179	0	1	1	2	1	1	2	2	4	2	0	0	1	1	3	1	1	0	1
2041710	0	1	1	5	1	1	2	2	4	1	0	0	1	1	1	4	0	1	1
2041711	1	4	1	2	1	2	2	2	4	4	0	0	1	1	3	4	1	1	1
2041712	1	4	1	2	1	4	2	2	4	4	0	0	1	1	3	4	1	1	1
2041713	0	4	1	2	1	1	2	1	4	4	0	0	1	1	1	4	0	1	1
2041714	1	5	1	2	1	2	1	1	4	3	0	0	1	1	4	4	1	1	1
2041715	1	5	1	2	1	2	2	1	4	1	0	0	1	1	4	4	1	1	1
2041716	1	5	1	2	1	2	2	2	4	1	0	0	1	1	4	4	1	1	1
2041717	1	4	1	3	1	5	3	2	4	1	1	1	1	1	1	1	0	0	0
2041718	0	4	1	1	1	1	2	2	4	2	0	0	1	1	4	4	1	1	1
2041719	0	3	1	2	1	1	2	2	4	1	0	0	1	1	3	1	1	0	1
2041720	1	5	1	2	1	2	2	2	4	1	0	0	1	1	4	1	1	0	1
2041721	1	4	1	1	1	2	2	2	4	1	0	0	1	1	3	4	1	1	1
2041722	1	2	1	1	1	2	2	2	4	1	0	0	1	1	3	4	1	1	1
2041723	0	2	1	3	1	1	2	2	4	1	0	0	1	1	1	1	0	0	0
2041724	0	5	1	1	1	1	2	1	4	1	0	0	1	1	3	4	1	1	1
2041725	1	5	1	1	1	2	2	2	4	2	0	0	1	1	1	4	0	1	1
2041726	1	2	1	2	1	2	2	1	4	1	0	0	1	1	3	1	1	0	1
2041727	1	5	1	1	1	2	2	2	4	2	0	0	1	1	3	1	1	0	1
2041728	1	3	1	2	1	2	3	2	4	1	0	0	1	1	1	1	0	0	0
2041729	1	2	1	2	1	2	2	2	4	3	0	0	1	1	3	4	1	1	1
2041730	0	4	1	2	1	2	1	1	4	3	0	0	1	1	4	4	1	1	1
2041731	1	1	1	2	1	2	2	1	4	3	0	0	1	1	1	1	0	0	0
2041732	1	2	1	5	1	2	2	1	4	4	0	0	1	1	2	1	1	0	1
2041733	1	2	1	3	1	2	2	1	4	4	0	0	1	1	2	1	1	0	1
2041734	1	4	1	2	1	2	1	1	4	4	0	0	1	1	3	1	1	0	1
2041735	0	3	1	2	1	1	2	2	4	1	0	0	1	1	2	4	1	1	1
204181	1	3	1	2	1	2	3	2	1	2	0	0	1	1	3	4	1	1	1
204182	1	4	1	2	1	2	1	2	1	1	0	0	1	1	3	1	1	0	1
204183	1	3	1	2	1	2	2	2	1	3	0	0	1	1	4	4	1	1	1
204184	1	4	1	2	1	2	2	1	1	2	0	0	1	1	4	4	1	1	1
204185	0	5	1	1	1	2	2	1	1	1	0	0	1	1	3	1	1	0	1
204186	0	5	1	1	1	2	2	1	2	1	0	0	1	1	3	4	1	1	1
204187	1	2	1	5	1	2	2	2	4	2	0	0	1	1	1	1	0	0	0
204188	1	1	1	4	1	2	2	1	1	2	0	0	1	1	3	1	1	0	1
204189	1	4	1	2	1	2	2	1	1	1	0	0	1	1	3	4	1	1	1
2041810	1	5	1	3	1	2	1	2	1	2	0	0	1	1	1	1	0	0	0
2041811	1	4	1	4	1	2	3	2	4	2	0	0	1	1	3	4	1	1	1
2041812	0	3	1	2	1	2	3	1	1	1	0	0	1	1	1	6	0	1	1
2041813	0	3	1	2	1	2	2	1	4	2	0	0	1	1	4	1	1	0	1
2041814	1	3	1	4	1	2	2	1	3	4	0	0	1	1	1	1	0	0	0
2041815	1	1	1	5	1	2	1	1	4	4	0	0	1	1	3	1	1	0	1
2041816	1	3	1	2	1	2	2	1	4	4	0	0	1	1	3	1	1	0	1
2041817	1	4	1	2	1	2	2	2	4	4	0	0	1	1	1	1	0	0	0
2041818	1	5	1	2	1	2	2	2	1	1	0	0	1	1	3	1	1	0	1
2041819	1	2	1	5	1	5	2	1	4	4	1	1	1	1	1	1	0	0	0
2041820	0	4	1	1	1	2	3	2	1	1	0	0	1	1	4	1	1	0	1
2041821	1	3	1	3	1	2	2	2	4	2	0	0	1	1	3	1	1	0	1
2041822	1	4	1	1	1	2	2	1	4	4	0	0	1	1	2	1	1	0	1
2041823	1	5	1	1	1	2	2	2	1	1	0	0	1	1	3	1	1	0	1
2041824	1	1	1	4	1	2	2	1	1	1	0	0	1	1	1	4	0	1	1

2041825	0	1	1	3	1	1	2	1	1	1	0	0	1	1	3	4	1	1	1
2041826	1	3	1	2	1	2	2	2	1	1	0	0	1	1	3	4	1	1	1
2041827	1	5	1	2	1	2	2	1	1	1	0	0	1	1	3	4	1	1	1
2041828	1	5	1	1	1	2	2	2	4	4	0	0	1	1	3	4	1	1	1
2041829	1	1	1	4	1	2	2	1	4	4	0	0	1	1	4	1	1	0	1
2041830	1	4	1	2	1	1	2	2	1	1	0	0	1	1	4	1	1	0	1
204191	1	2	1	4	1	2	2	2	3	4	0	0	1	1	1	5	0	1	1
204192	1	1	1	5	1	2	2	1	1	2	0	0	1	1	3	1	1	0	1
204193	0	2	1	2	1	2	1	1	3	4	0	0	1	1	3	1	1	0	1
204194	1	5	1	1	1	2	1	1	3	4	0	0	1	1	3	7	1	1	1
204195	0	3	1	2	1	2	2	2	1	3	0	0	1	1	1	5	0	1	1
204196	0	4	1	2	1	2	1	1	1	2	0	0	1	1	3	1	1	0	1
204197	0	5	1	1	1	2	1	1	1	2	0	0	1	1	2	1	1	0	1
204198	0	3	1	4	1	1	2	1	1	2	0	0	1	1	3	7	1	1	1
204199	0	5	1	1	1	2	1	1	1	4	0	0	0	99	99	99	99	99	99
2041910	0	3	1	2	1	2	1	2	3	4	0	0	1	1	3	5	1	1	1
2041911	0	3	1	2	1	2	2	2	1	2	0	0	1	1	2	7	1	1	1
2041912	1	2	1	3	1	2	2	1	3	4	0	0	1	1	3	1	1	0	1
2041913	1	5	1	1	1	2	1	1	3	4	0	0	1	1	3	6	1	1	1
2041914	1	5	1	1	1	2	1	1	3	4	0	0	1	1	3	1	1	0	1
2041915	0	3	1	2	1	2	1	1	3	2	0	0	1	1	3	1	1	0	1
2041916	1	2	1	2	1	2	2	1	3	4	0	0	1	1	3	1	1	0	1
2041917	1	2	1	2	1	2	1	2	3	4	0	0	1	1	2	1	1	0	1
2041918	1	3	1	2	1	2	2	2	3	3	0	0	1	1	3	1	1	0	1
2041919	1	3	1	2	1	2	1	1	3	4	0	0	1	1	3	1	1	0	1
2041920	0	5	1	1	1	2	1	1	3	4	0	0	1	1	2	1	1	0	1
2041921	0	3	1	2	1	2	2	2	3	4	0	0	1	1	1	1	0	0	0
2041922	1	4	1	2	1	2	1	1	3	3	0	0	1	1	2	1	1	0	1
2041923	1	5	1	1	1	2	1	1	3	4	0	0	1	1	2	6	1	1	1
2041924	1	2	1	2	1	2	2	2	3	4	0	0	1	1	3	1	1	0	1
2041925	1	2	1	2	1	2	1	1	3	4	0	0	1	1	1	1	0	0	0
2041926	0	2	1	3	1	2	1	1	3	4	0	0	1	1	1	1	0	0	0
2041927	1	4	1	1	1	2	2	2	3	4	0	0	1	1	2	1	1	0	1
2041928	0	4	1	1	1	2	1	1	3	4	0	0	1	1	1	1	0	0	0
2041929	0	1	1	4	1	2	1	1	3	4	0	0	1	1	2	1	1	0	1
2041930	1	2	1	2	1	2	1	1	1	4	0	0	1	1	2	1	1	0	1
204201	1	2	1	4	1	2	2	1	1	4	0	0	1	1	3	7	1	1	1
204202	0	1	1	4	1	2	2	1	1	4	0	0	1	1	3	1	1	0	1
204203	1	3	1	5	1	5	2	1	1	4	0	0	1	1	3	1	1	0	1
204204	1	2	1	2	1	2	1	1	1	4	0	0	1	1	3	7	1	1	1
204205	1	3	1	2	1	2	2	2	1	2	0	0	1	1	2	1	1	0	1
204206	1	5	1	1	1	2	1	1	1	2	0	0	0	99	99	99	99	99	99
204207	1	3	1	2	1	2	1	2	1	1	0	0	1	1	3	1	1	0	1
204208	1	1	1	3	1	2	2	1	1	2	0	0	1	1	3	1	1	0	1
204209	0	3	1	2	1	1	1	1	1	2	0	0	1	1	2	1	1	0	1
2042010	0	3	1	2	1	3	1	2	1	2	0	0	1	1	3	1	1	0	1
2042011	1	5	1	1	1	2	1	1	1	3	0	0	1	1	2	1	1	0	1
2042012	0	5	1	1	1	2	1	1	1	3	0	0	0	99	99	99	99	99	99
2042013	1	3	1	2	1	2	2	2	1	4	0	0	1	1	3	1	1	0	1
2042014	0	4	1	1	1	2	1	2	1	4	0	0	1	1	2	1	1	0	1
2042015	1	2	1	2	1	2	2	1	1	2	0	0	1	1	2	1	1	0	1
2042016	0	2	1	2	1	1	1	1	1	2	0	0	1	1	3	6	1	1	1
2042017	0	2	1	2	1	2	3	2	1	1	0	0	1	1	3	6	1	1	1
2042018	1	4	1	2	1	2	2	2	4	4	0	0	1	1	3	1	1	0	1
2042019	1	3	1	2	1	2	2	2	1	4	0	0	1	1	3	1	1	0	1
2042020	0	5	1	1	1	2	1	1	1	4	0	0	1	1	3	1	1	0	1

2042021	1	3	1	2	1	2	2	1	1	4	0	0	1	1	4	1	1	0	1
2042022	1	5	1	1	1	2	1	1	1	4	0	0	1	1	2	6	1	1	1
2042023	1	2	1	2	1	2	2	2	1	4	0	0	1	1	1	1	0	0	0
2042024	1	1	1	4	1	2	2	1	1	4	0	0	1	1	4	1	1	0	1
2042025	1	3	1	2	1	2	1	2	1	4	0	0	1	1	3	1	1	0	1
205211	0	3	1	2	1	2	1	2	3	1	0	0	1	1	4	4	1	1	1
205212	1	3	1	2	1	2	3	2	3	1	0	0	1	1	1	1	0	0	0
205213	0	5	1	2	1	2	1	2	3	1	0	0	1	1	2	1	1	0	1
205214	0	3	1	2	1	2	1	1	3	2	0	0	1	1	2	1	1	0	1
205215	1	4	1	4	1	2	1	1	3	3	0	0	1	1	2	1	1	0	1
205216	0	4	1	1	1	2	1	1	3	3	0	0	1	1	3	1	1	0	1
205217	0	2	1	2	1	2	1	2	3	3	0	0	1	1	2	1	1	0	1
205218	1	2	1	4	1	2	1	2	3	3	0	0	1	1	1	1	0	0	0
205219	1	5	1	2	1	2	1	2	3	4	0	0	1	1	1	1	0	0	0
2052110	0	3	1	2	1	2	2	2	3	2	0	0	1	1	1	1	0	0	0
2052111	0	4	1	2	1	2	1	1	3	1	0	0	1	1	2	1	1	0	1
2052112	1	3	1	2	1	2	2	1	3	3	0	0	1	1	2	1	1	0	1
2052113	0	3	1	1	1	2	2	1	3	2	0	0	1	1	2	1	1	0	1
2052114	0	2	1	2	1	2	1	2	3	2	0	0	1	1	1	1	0	0	0
2052115	0	1	1	5	1	5	2	1	3	1	0	0	1	1	1	1	0	0	0
2052116	1	3	1	2	1	2	1	2	3	4	0	0	1	1	3	1	1	0	1
2052117	1	2	1	2	1	2	2	1	3	1	0	0	1	1	1	1	0	0	0
2052118	0	3	1	2	1	2	1	2	3	1	0	0	1	1	5	4	1	1	1
2052119	0	3	1	2	1	2	1	2	3	1	0	0	1	1	1	1	0	0	0
2052120	1	5	1	1	1	2	1	1	3	1	0	0	1	1	2	1	1	0	1
2052121	0	3	1	2	1	2	2	2	3	1	0	0	1	1	3	1	1	0	1
2052122	1	3	1	2	1	2	1	2	1	1	0	0	0	99	99	99	99	99	99
2052123	0	3	1	2	1	2	2	2	3	1	0	0	1	1	1	1	0	0	0
2052124	0	3	1	2	1	2	1	2	3	1	0	0	1	1	2	1	1	0	1
2052125	1	2	1	2	1	2	1	2	1	4	0	0	1	1	2	1	1	0	1
2052126	1	2	1	4	1	2	2	2	3	2	0	0	1	1	1	1	0	0	0
2052127	0	3	1	2	1	2	1	2	3	2	0	0	1	1	2	1	1	0	1
2052128	0	3	1	3	2	2	2	2	3	2	0	0	1	1	1	1	0	0	0
2052129	1	4	1	2	1	2	2	1	2	1	0	0	1	1	3	1	1	0	1
2052130	0	5	1	1	1	2	1	1	3	3	0	0	0	99	99	99	99	99	99
205221	0	2	1	1	1	2	2	2	4	4	0	0	1	1	1	1	0	0	0
205222	1	3	1	1	1	2	2	2	4	4	0	0	1	1	2	1	1	0	1
205223	1	1	1	1	1	2	1	2	4	4	0	0	1	1	3	1	1	0	1
205224	0	1	1	1	2	3	2	1	4	4	0	0	1	1	2	7	1	1	1
205225	1	2	1	3	1	2	1	2	4	4	0	0	1	1	3	1	1	0	1
205226	1	3	1	2	1	2	1	1	4	4	0	0	1	1	3	1	1	0	1
205227	0	4	1	1	1	2	2	2	4	4	0	0	1	1	3	1	1	0	1
205228	0	4	1	1	1	2	1	2	3	4	0	0	1	1	2	1	1	0	1
205229	0	1	1	4	1	2	2	2	3	2	0	0	1	1	2	1	1	0	1
2052210	1	5	1	1	1	4	2	2	3	1	0	0	1	1	2	1	1	0	1
2052211	0	3	1	2	1	2	2	2	3	1	0	0	1	1	1	1	0	0	0
2052212	0	1	1	4	1	2	2	1	3	1	0	0	1	1	1	1	0	0	0
2052213	0	4	1	3	2	1	2	2	3	2	0	0	1	1	2	1	1	0	1
2052214	0	4	1	2	2	1	2	2	3	2	0	0	1	1	2	1	1	0	1
2052215	0	3	1	3	1	2	2	2	3	1	0	0	1	1	1	1	0	0	0
2052216	0	2	1	1	1	2	1	2	3	2	0	0	1	1	1	2	0	0	0
2052217	0	2	1	2	1	2	1	2	3	3	0	0	1	1	1	1	0	0	0
2052218	0	1	1	2	1	2	1	1	3	4	0	0	1	1	2	1	1	0	1
2052219	0	5	1	1	1	2	1	2	3	4	0	0	1	1	2	1	1	0	1
2052220	0	2	1	2	1	2	2	2	3	4	0	0	1	1	3	1	1	0	1
2052221	0	3	1	2	1	2	2	2	3	4	0	0	1	1	3	1	1	0	1

2052222	0	2	1	1	1	3	3	2	2	4	0	0	1	1	2	1	1	0	1
2052223	0	2	1	1	1	2	1	2	3	4	0	0	1	1	2	1	1	0	1
2052224	1	5	1	2	1	2	2	1	3	4	0	0	1	1	2	1	1	0	1
2052225	0	3	1	1	1	2	2	1	3	1	0	0	1	1	2	1	1	0	1
2052226	0	4	1	1	1	1	2	2	3	1	0	0	1	1	1	1	0	0	0
2052227	0	2	1	1	1	1	1	2	3	1	0	0	1	1	1	1	0	0	0
2052228	0	1	1	1	1	2	1	2	4	2	0	0	1	1	2	1	1	0	1
2052229	0	5	1	3	2	2	2	1	4	2	0	0	1	1	1	1	0	0	0
2052230	0	2	1	1	1	2	1	2	3	1	0	0	1	1	2	1	1	0	1
205231	0	1	1	5	1	5	2	1	3	1	0	0	1	1	2	1	1	0	1
205232	0	3	1	1	1	1	2	2	3	1	0	0	1	1	3	1	1	0	1
205233	0	1	1	4	1	2	2	2	3	1	0	0	1	1	1	1	0	0	0
205234	0	3	1	4	1	2	2	1	3	1	0	0	1	1	2	1	1	0	1
205235	1	2	1	3	1	3	3	1	3	1	1	0	1	1	4	1	1	0	1
205236	0	3	1	2	1	2	2	1	3	1	0	0	1	1	2	1	1	0	1
205237	0	2	1	2	1	3	2	2	4	2	0	0	1	1	1	1	0	0	0
205238	1	3	1	1	1	2	2	2	4	3	0	0	1	1	2	1	1	0	1
205239	0	1	1	3	2	1	1	2	4	4	0	0	1	1	3	1	1	0	1
2052310	1	3	1	1	1	2	2	2	4	4	0	0	1	1	3	1	1	0	1
2052311	1	2	1	1	1	4	1	2	4	2	0	0	1	1	2	1	1	0	1
2052312	1	3	1	1	1	4	2	2	4	3	0	0	1	1	2	1	1	0	1
2052313	0	3	1	2	1	2	2	1	4	4	0	0	1	1	2	1	1	0	1
2052314	0	3	1	5	1	5	2	2	4	4	0	0	1	1	1	1	0	0	0
2052315	0	2	1	1	1	2	2	2	4	2	0	0	1	1	2	1	1	0	1
2052316	1	5	1	1	1	2	2	2	4	3	0	0	1	1	3	1	1	0	1
2052317	0	1	1	1	1	2	2	1	4	4	0	0	1	1	3	1	1	0	1
2052318	1	5	1	4	1	5	3	2	4	4	1	1	1	1	1	1	0	0	0
2052319	1	5	1	5	1	5	3	2	4	1	0	0	1	1	1	1	0	0	0
2052320	0	2	1	2	1	3	3	2	3	1	1	1	1	1	2	1	1	0	1
2052321	1	5	1	4	1	2	2	2	3	1	0	0	1	1	2	1	1	0	1
2052322	0	4	1	1	1	2	2	2	3	1	0	0	1	1	3	1	1	0	1
2052323	1	5	1	1	1	2	2	2	3	1	0	0	1	1	4	1	1	0	1
2052324	0	1	1	1	1	2	1	2	3	1	0	0	1	1	2	1	1	0	1
2052325	1	3	1	1	1	2	2	2	3	1	0	0	1	1	3	1	1	0	1
2052326	0	1	1	2	1	2	2	2	3	1	0	0	1	1	3	1	1	0	1
2052327	1	5	1	1	1	2	1	1	3	1	0	0	1	1	2	1	1	0	1
2052328	1	3	1	2	1	2	2	2	3	1	0	0	1	1	1	1	0	0	0
2052329	1	1	1	1	1	2	2	1	4	1	0	0	1	1	2	6	1	1	1
2052330	0	1	1	1	1	1	1	2	4	1	0	0	1	1	1	1	0	0	0
205241	0	1	1	4	2	1	2	1	4	4	0	0	1	1	1	1	0	0	0
205242	0	3	1	2	1	2	3	2	4	3	0	0	1	1	1	6	0	1	1
205243	0	3	1	4	2	1	3	1	4	4	1	1	1	1	1	1	0	0	0
205244	0	3	1	3	2	1	3	2	4	3	1	1	1	1	1	1	0	0	0
205245	1	3	1	4	2	2	2	2	4	4	1	1	1	1	1	1	0	0	0
205246	0	4	1	5	2	5	3	2	4	4	1	1	1	1	1	1	0	0	0
205247	1	4	1	3	1	2	3	2	4	4	0	0	1	1	1	1	0	0	0
205248	1	3	1	2	1	4	2	2	4	4	0	0	1	1	2	5	1	1	1
205249	0	2	1	2	2	1	2	2	4	4	0	0	1	1	3	1	1	0	1
2052410	0	2	1	2	1	1	3	2	4	4	0	0	1	1	3	1	1	0	1
2052411	1	2	1	1	1	4	3	1	4	4	1	0	1	1	1	1	0	0	0
2052412	0	4	1	3	2	3	3	1	4	4	1	1	1	1	1	1	0	0	0
2052413	1	5	1	3	1	4	2	2	4	4	0	0	1	1	1	1	0	0	0
2052414	1	4	1	2	2	2	3	2	4	4	0	0	1	1	3	1	1	0	1
2052415	0	3	1	4	1	5	3	2	4	2	1	1	1	1	1	1	0	0	0
2052416	1	4	1	1	1	2	2	2	4	3	0	0	1	1	1	6	0	1	1
2052417	0	3	1	5	2	5	3	1	4	3	0	0	1	1	1	1	0	0	0

2052418	1	3	1	1	1	5	2	2	4	4	0	0	1	1	3	1	1	0	1
2052419	0	1	1	1	1	1	2	2	4	2	0	0	1	1	2	1	1	0	1
2052420	0	2	1	1	1	1	3	2	4	3	1	1	1	1	3	1	1	0	1
2052421	1	5	1	1	1	2	2	2	4	4	0	0	1	1	2	1	1	0	1
2052422	1	1	1	3	2	5	3	2	4	4	0	0	1	1	1	1	0	0	0
2052423	0	1	1	4	2	1	2	1	4	4	0	0	1	1	3	6	1	1	1
2052424	0	2	1	2	2	1	2	1	4	4	0	0	1	1	1	1	0	0	0
2052425	1	3	1	2	1	4	3	2	4	4	0	0	1	1	2	1	1	0	1
2052426	1	3	1	2	1	4	3	2	4	4	0	0	1	1	1	1	0	0	0
2052427	0	2	1	3	1	1	3	1	4	4	1	1	1	1	2	1	1	0	1
2052428	1	3	1	4	2	4	3	1	4	4	1	1	1	1	3	1	1	0	1
2052429	0	4	1	1	1	1	3	2	4	2	0	0	1	1	3	1	1	0	1
2052430	0	5	1	1	2	3	2	1	4	4	0	0	1	1	4	1	1	0	1
205251	0	2	1	2	1	2	2	1	2	1	0	0	1	1	1	1	0	0	0
205252	0	3	1	1	1	2	1	1	2	1	0	0	1	1	3	1	1	0	1
205253	0	2	1	4	1	1	2	2	2	1	0	0	1	1	2	1	1	0	1
205254	1	5	1	2	1	2	2	1	2	1	0	0	1	1	3	1	1	0	1
205255	0	2	1	1	1	3	1	2	2	1	0	0	1	1	2	1	1	0	1
205256	0	2	1	2	1	1	2	2	2	1	0	0	1	1	2	1	1	0	1
205257	0	2	1	2	1	2	2	2	2	1	0	0	1	1	3	1	1	0	1
205258	1	2	1	1	1	2	1	1	2	1	0	0	1	1	3	1	1	0	1
205259	0	3	1	1	2	2	1	2	2	1	0	0	1	1	1	1	0	0	0
2052510	1	5	1	1	1	2	1	1	2	1	0	0	1	1	3	1	1	0	1
2052511	1	2	1	2	1	2	1	2	2	1	0	0	1	1	3	1	1	0	1
2052512	0	1	1	2	1	2	1	2	2	1	0	0	1	1	2	1	1	0	1
2052513	0	2	1	1	1	2	1	2	2	1	0	0	1	1	2	1	1	0	1
2052514	0	2	1	4	1	2	2	2	2	1	0	0	1	1	2	1	1	0	1
2052515	0	2	1	2	1	2	2	2	2	1	1	0	1	1	3	1	1	0	1
2052516	0	2	1	4	1	3	2	2	2	1	0	0	1	1	1	1	0	0	0
2052517	0	2	1	1	1	2	1	1	2	2	0	0	1	1	2	1	1	0	1
2052518	0	2	1	1	1	2	1	2	2	2	0	0	1	1	2	1	1	0	1
2052519	1	2	1	1	1	2	1	2	2	1	0	0	1	1	3	1	1	0	1
2052520	0	1	1	1	1	2	2	2	2	1	0	0	1	1	2	1	1	0	1
2052521	0	3	1	1	1	2	1	2	3	1	0	0	1	1	2	1	1	0	1
2052522	0	2	1	1	1	2	2	2	2	1	0	0	1	1	2	1	1	0	1
2052523	0	3	1	1	1	2	2	2	3	1	0	0	1	1	3	1	1	0	1
2052524	0	3	1	1	1	2	2	2	3	1	0	0	1	1	2	1	1	0	1
2052525	0	3	1	2	1	3	3	2	3	2	0	0	1	1	2	1	1	0	1
2052526	0	2	1	2	1	2	1	2	3	2	0	0	1	1	2	1	1	0	1
2052527	0	1	1	1	1	1	2	1	3	3	0	0	1	1	2	1	1	0	1
2052528	1	3	1	1	1	2	2	2	3	3	0	0	1	1	2	1	1	0	1
2052529	0	3	1	1	1	2	1	2	2	1	0	0	1	1	1	1	0	0	0
2052530	1	3	1	3	1	3	2	2	2	2	0	0	1	1	2	1	1	0	1
206261	1	3	1	2	1	2	2	1	3	2	0	0	1	1	1	1	0	0	0
206262	1	1	1	2	1	2	1	1	3	2	0	0	1	1	3	1	1	0	1
206263	1	3	1	3	1	2	2	2	3	1	0	0	1	1	1	1	0	0	0
206264	1	2	1	4	1	2	2	2	3	1	0	0	1	1	1	1	0	0	0
206265	1	4	1	2	1	2	2	2	3	1	0	0	1	1	1	1	0	0	0
206266	0	1	1	2	1	2	1	1	3	3	0	0	1	1	3	1	1	0	1
206267	1	2	1	3	1	2	2	1	3	2	0	0	1	1	1	1	0	0	0
206268	0	1	1	4	1	1	2	2	3	3	0	0	1	1	1	1	0	0	0
206269	0	4	1	2	1	2	2	2	3	4	0	0	1	1	1	1	0	0	0
2062610	0	1	1	2	1	2	2	2	3	4	0	0	1	1	3	1	1	0	1
2062611	0	2	1	1	1	2	1	1	3	4	0	0	1	1	1	1	0	0	0
2062612	0	1	1	4	1	2	1	2	3	4	0	0	1	1	1	1	0	0	0
2062613	0	3	1	1	1	2	2	2	3	4	0	0	1	1	3	1	1	0	1

2062614	0	3	1	2	1	2	2	1	3	4	1	1	1	1	1	1	0	0	0
2062615	1	4	1	1	1	2	1	1	3	3	0	0	1	1	3	1	1	0	1
2062616	1	3	1	1	1	2	1	1	3	4	0	0	1	1	3	1	1	0	1
2062617	0	1	1	3	1	5	1	2	3	3	0	0	1	1	2	1	1	0	1
2062618	1	5	1	1	1	2	2	2	3	3	0	0	1	1	2	1	1	0	1
2062619	0	2	1	2	1	2	2	2	3	4	0	0	1	1	3	1	1	0	1
2062620	0	2	1	1	1	2	2	2	3	2	0	0	1	1	2	1	1	0	1
2062621	0	2	1	1	1	1	1	2	3	2	0	0	1	1	1	1	0	0	0
2062622	0	2	1	1	1	3	3	2	3	1	0	0	1	1	1	1	0	0	0
2062623	0	4	1	1	1	2	2	1	3	2	0	0	1	1	3	1	1	0	1
2062624	1	3	1	2	1	2	2	2	3	1	0	0	1	1	1	1	0	0	0
2062625	1	3	1	3	1	2	2	1	3	2	0	0	1	1	3	1	1	0	1
206271	0	4	1	1	1	2	1	2	3	1	0	0	1	1	2	1	1	0	1
206272	0	1	1	4	1	2	3	1	3	1	0	0	1	1	2	1	1	0	1
206273	0	2	1	2	1	2	2	2	3	1	0	0	1	1	2	1	1	0	1
206274	0	1	1	2	1	1	3	1	3	1	0	0	1	1	2	1	1	0	1
206275	1	1	1	1	1	2	1	2	3	1	0	0	1	1	2	1	1	0	1
206276	0	3	1	2	1	2	2	2	3	4	0	0	1	1	2	1	1	0	1
206277	1	1	1	4	1	2	2	1	3	4	0	0	1	1	1	1	0	0	0
206278	0	2	1	4	1	2	2	1	3	4	0	0	1	1	2	1	1	0	1
206279	1	3	1	2	1	2	1	2	3	4	0	0	1	1	1	1	0	0	0
2062710	0	2	1	2	1	2	1	1	3	4	0	0	1	1	1	1	0	0	0
2062711	0	3	1	1	1	2	1	2	1	2	0	0	1	1	2	1	1	0	1
2062712	0	2	1	1	1	2	2	1	3	4	0	0	1	1	1	1	0	0	0
2062713	1	1	1	3	1	2	2	2	3	4	0	0	1	1	1	1	0	0	0
2062714	1	1	1	2	1	2	2	1	3	4	0	0	1	1	2	1	1	0	1
2062715	1	1	1	1	1	2	2	2	3	4	0	0	1	1	1	1	0	0	0
2062716	1	2	1	3	1	2	1	2	3	4	0	0	1	1	1	1	0	0	0
2062717	0	1	1	3	1	2	2	1	3	4	0	0	1	1	2	1	1	0	1
2062718	1	1	1	1	1	2	1	1	3	4	0	0	1	1	2	1	1	0	1
2062719	0	2	1	2	1	2	2	1	3	4	0	0	1	1	2	1	1	0	1
2062720	0	1	1	3	1	1	2	1	3	4	0	0	1	1	2	1	1	0	1
2062721	1	4	1	1	1	2	1	2	3	4	0	0	1	1	2	1	1	0	1
2062722	0	2	1	3	1	2	1	2	3	4	0	0	1	1	1	1	0	0	0
2062723	1	3	1	2	1	2	1	2	3	4	0	0	1	1	1	1	0	0	0
2062724	0	2	1	2	1	2	2	2	3	4	0	0	1	1	1	1	0	0	0
2062725	1	2	1	2	1	2	2	2	3	4	0	0	1	1	1	1	0	0	0
2062726	1	1	1	1	1	2	1	1	1	2	0	0	1	1	4	5	1	1	1
2062727	1	3	1	1	1	2	2	2	3	2	0	0	1	1	3	6	1	1	1
2062728	0	1	1	2	1	2	2	2	3	2	0	0	1	1	2	1	1	0	1
2062729	0	3	1	1	1	2	2	2	3	2	0	0	1	1	2	1	1	0	1
2062730	0	2	1	2	1	1	1	2	3	2	0	0	1	1	2	1	1	0	1
206281	0	3	1	3	1	1	2	2	3	3	0	0	1	1	2	2	1	0	1
206282	1	2	1	4	1	2	2	2	3	2	0	0	1	1	5	5	1	1	1
206283	1	3	1	2	1	2	2	2	3	1	0	0	1	1	1	1	0	0	0
206284	1	3	1	2	1	2	2	2	3	1	0	0	1	1	2	1	1	0	1
206285	0	2	1	2	1	3	2	2	3	1	0	0	1	1	3	1	1	0	1
206286	0	5	1	2	1	1	2	2	3	1	0	0	1	1	2	1	1	0	1
206287	0	2	1	2	1	3	2	2	3	1	0	0	1	1	2	1	1	0	1
206288	0	2	1	1	1	1	2	2	2	4	0	0	1	1	2	1	1	0	1
206289	0	1	1	4	1	1	2	2	2	4	0	0	1	1	4	1	1	0	1
2062810	1	2	1	1	1	2	2	2	2	4	0	0	1	1	2	1	1	0	1
2062811	1	4	1	1	1	2	1	2	2	4	0	0	1	0	5	5	1	1	1
2062812	1	2	1	1	1	2	1	2	2	4	0	0	1	1	4	2	1	0	1
2062813	0	1	1	3	1	3	1	1	2	4	0	0	1	1	2	1	1	0	1
2062814	1	4	1	4	2	2	2	2	1	1	0	0	1	1	4	2	1	0	1

2062815	0	3	1	2	1	1	1	2	1	3	0	0	1	1	5	5	1	1	1
2062816	1	1	1	2	2	2	2	1	1	2	0	0	1	1	2	2	1	0	1
2062817	0	4	1	2	1	1	2	2	1	1	0	0	1	1	1	1	0	0	0
2062818	0	3	1	1	1	1	2	2	2	4	0	0	1	1	4	1	1	0	1
2062819	0	4	1	2	2	5	2	2	1	2	0	0	1	1	2	1	1	0	1
2062820	1	4	1	1	2	2	3	2	1	4	0	0	1	0	5	5	1	1	1
2062821	0	3	1	1	2	2	2	2	2	1	0	0	1	1	3	2	1	0	1
2062822	1	3	1	2	2	2	3	2	1	2	0	0	1	1	1	6	0	1	1
2062823	1	3	1	1	1	2	3	2	1	1	0	0	1	1	2	1	1	0	1
2062824	0	3	1	1	2	2	2	2	1	1	0	0	1	1	1	1	0	0	0
2062825	0	2	1	2	2	1	2	2	1	1	0	0	1	1	1	1	0	0	0
2062826	1	3	1	1	2	2	1	1	1	1	0	0	1	1	3	5	1	1	1
2062827	1	3	1	4	1	3	3	2	1	2	0	0	1	1	5	7	1	1	1
2062828	1	5	1	2	1	2	2	1	3	3	0	0	1	1	2	2	1	0	1
2062829	1	5	1	1	1	2	2	2	3	1	0	0	1	1	3	1	1	0	1
2062830	1	4	1	4	2	2	3	2	1	1	0	0	1	1	1	1	0	0	0
206291	1	5	1	1	1	2	2	1	1	1	0	0	1	1	2	1	1	0	1
206292	0	3	1	2	1	2	2	2	1	1	0	0	1	1	1	1	0	0	0
206293	0	3	1	1	1	3	2	1	1	2	0	0	1	1	1	1	0	0	0
206294	1	2	1	2	1	2	2	2	1	3	0	0	1	1	2	1	1	0	1
206295	1	5	1	1	1	2	2	2	1	2	1	0	1	1	2	1	1	0	1
206296	0	4	1	1	1	2	2	2	1	3	0	0	1	1	4	1	1	0	1
206297	1	5	1	1	1	2	2	2	1	4	0	0	1	1	2	1	1	0	1
206298	1	5	1	1	1	2	1	1	1	4	0	0	0	99	99	99	99	99	99
206299	0	4	1	2	1	2	3	2	1	4	0	0	1	1	1	1	0	0	0
2062910	1	5	1	1	1	2	3	1	1	4	0	0	0	99	99	99	99	99	99
2062911	0	2	1	2	2	1	2	2	1	4	1	1	1	1	2	1	1	0	1
2062912	0	3	1	1	2	2	2	2	1	4	0	0	1	1	2	1	1	0	1
2062913	0	2	1	1	1	2	2	2	1	1	0	0	1	1	1	1	0	0	0
2062914	0	3	1	1	1	2	2	2	1	1	0	0	1	1	2	1	1	0	1
2062915	1	1	1	5	1	4	2	2	1	1	1	1	1	1	1	1	0	0	0
2062916	1	2	1	4	1	5	3	2	1	1	1	1	1	1	2	1	1	0	1
2062917	1	5	1	1	1	2	2	2	1	3	0	0	1	1	1	1	0	0	0
2062918	0	2	1	3	1	1	2	2	1	2	0	0	1	1	2	1	1	0	1
2062919	0	3	1	1	1	1	2	2	1	1	0	0	1	1	2	4	1	1	1
2062920	0	1	1	4	1	3	3	2	1	1	0	0	1	1	2	6	1	1	1
2062921	1	3	1	2	1	2	3	2	1	1	0	0	1	1	1	1	0	0	0
2062922	0	2	1	1	1	1	2	2	1	2	0	0	1	1	3	1	1	0	1
2062923	0	3	1	4	1	1	2	2	1	3	0	0	1	1	2	1	1	0	1
2062924	1	4	1	1	1	2	3	2	1	1	0	0	1	1	3	6	1	1	1
2062925	1	2	1	1	1	2	2	2	1	1	0	0	1	1	2	1	1	0	1
2062926	0	2	1	2	1	1	2	2	1	1	0	0	1	1	2	5	1	1	1
2062927	0	2	1	2	1	1	2	2	1	1	0	0	1	1	1	2	0	0	0
2062928	0	2	1	1	1	1	2	2	1	2	0	0	1	1	1	1	0	0	0
2062929	0	2	1	1	1	1	2	2	1	3	0	0	1	1	2	1	1	0	1
2062930	1	4	1	2	1	2	2	2	1	3	0	0	1	1	2	2	1	0	1
206301	0	5	1	2	1	3	2	1	1	1	0	0	1	1	1	1	0	0	0
206302	0	4	1	1	1	3	2	1	1	2	0	0	1	1	1	1	0	0	0
206303	0	5	1	4	2	1	1	1	1	1	0	0	0	99	99	99	99	99	99
206304	0	1	1	4	2	1	2	1	1	4	1	1	1	1	1	1	0	0	0
206305	0	5	1	1	1	3	2	1	1	2	0	0	0	99	99	99	99	99	99
206306	1	4	1	3	1	2	2	2	1	1	0	0	1	1	1	1	0	0	0
206307	1	3	1	3	1	4	2	1	1	1	0	0	1	1	1	1	0	0	0
206308	0	5	1	1	1	1	2	1	1	3	0	0	0	99	99	99	99	99	99
206309	0	2	1	5	1	5	2	1	1	1	1	1	1	1	1	1	0	0	0
2063010	1	3	1	1	2	4	3	1	1	3	1	0	1	1	1	1	0	0	0

2063011	1	4	1	2	1	2	3	1	1	1	1	1	1	1	1	1	0	0	0
2063012	0	2	1	2	2	1	2	1	1	1	0	0	1	1	1	1	0	0	0
2063013	0	1	1	5	2	1	3	1	1	4	0	0	1	1	1	1	0	0	0
2063014	0	5	1	4	2	3	3	1	1	2	1	1	1	1	1	1	0	0	0
2063015	1	5	1	1	2	2	2	1	1	3	0	0	0	99	99	99	99	99	99
2063016	0	3	1	2	1	1	2	1	1	4	0	0	1	1	1	1	0	0	0
2063017	1	4	1	3	1	4	3	1	1	4	1	1	1	1	1	1	0	0	0
2063018	0	3	1	4	1	1	2	1	1	3	1	0	1	1	1	1	0	0	0
2063019	0	3	1	2	2	1	1	1	1	1	0	0	1	1	1	5	0	1	1
2063020	0	2	1	2	2	3	3	1	1	1	1	0	1	1	1	1	0	0	0
2063021	0	1	1	2	1	3	2	1	1	4	0	0	1	1	1	1	0	0	0
2063022	1	2	1	2	1	2	2	1	1	1	0	0	1	1	1	1	0	0	0
2063023	1	2	1	2	1	2	2	1	1	1	0	0	1	1	1	1	0	0	0
2063024	1	3	1	2	1	2	2	1	1	3	0	0	1	1	1	5	0	1	1
2063025	0	5	1	2	1	2	2	1	1	1	0	0	0	99	99	99	99	99	99
206311	0	1	1	2	1	1	2	1	4	4	0	0	1	1	1	1	0	0	0
206312	0	2	1	3	1	1	2	1	4	4	0	0	1	1	1	1	0	0	0
206313	1	2	1	2	1	2	2	2	4	3	0	0	0	99	99	99	99	99	99
206314	1	3	1	1	1	2	2	1	4	4	0	0	1	1	1	1	0	0	0
206315	0	2	1	2	1	1	2	1	4	2	1	0	1	1	1	1	0	0	0
206316	0	1	1	5	1	1	3	1	4	4	0	0	1	1	1	1	0	0	0
206317	0	2	1	1	1	3	2	1	4	4	1	0	1	1	1	1	0	0	0
206318	1	3	1	1	1	2	2	1	4	1	1	0	1	1	1	1	0	0	0
206319	0	5	1	1	1	1	1	1	4	1	0	0	0	99	99	99	99	99	99
2063110	1	2	1	2	1	2	2	1	4	3	1	0	1	1	1	1	0	0	0
2063111	0	3	1	1	1	3	2	1	4	4	0	0	1	1	1	1	0	0	0
2063112	0	2	1	1	1	1	2	1	4	3	0	0	1	1	1	1	0	0	0
2063113	1	2	1	2	1	2	2	1	4	2	0	0	1	1	1	1	0	0	0
2063114	1	3	1	2	1	2	2	1	4	2	1	0	1	1	1	1	0	0	0
2063115	0	3	1	2	1	1	2	1	4	4	0	0	0	99	99	99	99	99	99
2063116	1	2	1	2	1	2	2	1	4	3	1	0	1	1	1	1	0	0	0
2063117	0	3	1	3	2	1	2	1	4	4	0	0	1	1	1	1	0	0	0
2063118	1	3	1	2	1	3	2	1	4	3	0	0	1	1	4	1	1	0	1
2063119	0	2	1	2	1	1	2	1	4	3	0	0	1	1	1	1	0	0	0
2063120	1	1	1	2	1	2	2	1	4	1	0	0	1	1	1	1	0	0	0
2063121	1	2	1	2	1	2	2	1	4	4	0	0	1	1	1	1	0	0	0
2063122	1	4	1	1	1	2	2	1	4	3	0	0	1	1	1	1	0	0	0
2063123	0	2	1	1	1	1	2	1	4	3	0	0	1	1	1	1	0	0	0
2063124	0	1	1	1	1	1	3	1	4	1	0	0	1	1	1	1	0	0	0
2063125	1	2	1	2	1	2	3	2	4	4	0	0	1	1	1	1	0	0	0
206321	0	2	1	1	1	1	1	2	4	4	0	0	1	1	2	1	1	0	1
206322	0	2	1	1	1	3	1	1	4	4	0	0	1	1	4	1	1	0	1
206323	0	1	1	2	1	1	2	1	4	4	0	0	1	1	3	1	1	0	1
206324	1	4	1	2	1	2	1	1	4	4	0	0	1	1	2	1	1	0	1
206325	0	1	1	3	1	1	2	1	4	4	0	0	1	1	3	1	1	0	1
206326	0	2	1	1	1	2	1	2	4	4	0	0	1	1	3	1	1	0	1
206327	0	5	1	1	1	2	1	2	4	4	0	0	1	1	3	1	1	0	1
206328	0	3	1	1	1	1	1	2	4	4	0	0	1	1	3	2	1	0	1
206329	0	1	1	3	1	1	3	2	1	1	0	0	1	1	3	1	1	0	1
2063210	0	1	1	3	1	3	1	2	1	1	0	0	1	1	2	1	1	0	1
2063211	1	4	1	1	1	2	1	2	1	1	0	0	1	1	3	1	1	0	1
2063212	1	3	1	2	1	2	2	2	1	1	1	1	1	1	3	1	1	0	1
2063213	0	3	1	1	1	2	2	2	1	1	0	0	1	1	2	1	1	0	1
2063214	0	2	1	2	2	1	2	2	4	4	0	0	1	1	2	1	1	0	1
2063215	0	1	1	3	1	5	2	2	4	4	0	0	1	1	2	1	1	0	1
2063216	0	4	1	1	1	3	2	1	4	4	0	0	1	1	2	1	1	0	1

2063217	1	2	1	2	1	2	2	2	1	2	0	0	1	1	1	1	0	0	0
2063218	1	1	1	4	1	2	2	2	1	2	0	0	1	1	2	1	1	0	1
2063219	0	5	1	2	1	2	2	2	1	1	0	0	1	1	3	1	1	0	1
2063220	0	2	1	1	1	1	2	2	1	1	0	0	1	1	3	1	1	0	1
2063221	1	2	1	2	1	2	2	1	1	4	0	0	1	1	2	1	1	0	1
2063222	0	3	1	2	1	1	3	2	1	1	0	0	1	1	3	1	1	0	1
2063223	0	2	1	2	1	1	2	2	4	1	0	0	1	1	4	1	1	0	1
2063224	0	5	1	1	1	3	2	1	1	1	0	0	1	1	4	1	1	0	1
2063225	0	3	1	1	1	3	1	2	4	4	0	0	1	1	2	4	1	1	1
2063226	1	2	1	2	1	2	1	1	1	2	0	0	1	1	2	1	1	0	1
2063227	1	3	1	3	1	2	2	2	1	2	0	0	1	1	2	1	1	0	1
2063228	1	1	1	3	1	2	2	2	1	2	0	0	1	1	3	1	1	0	1
2063229	0	1	1	4	1	1	3	2	1	1	0	0	1	1	1	1	0	0	0
2063230	1	4	1	1	1	2	1	1	4	4	0	0	1	1	5	5	1	1	1
206331	1	3	1	1	1	2	1	1	3	4	0	0	1	1	3	1	1	0	1
206332	1	3	1	1	1	2	1	2	3	4	0	0	1	1	3	1	1	0	1
206333	0	2	1	2	1	2	1	2	3	4	0	0	1	1	2	1	1	0	1
206334	1	5	1	3	1	2	1	1	3	3	0	0	1	0	5	5	1	1	1
206335	1	3	1	2	1	2	1	2	1	2	0	0	1	1	1	1	0	0	0
206336	1	2	1	2	1	2	2	2	1	1	0	0	1	1	1	1	0	0	0
206337	0	3	1	1	1	2	1	2	1	1	0	0	1	1	1	1	0	0	0
206338	0	4	1	2	1	1	1	2	1	1	0	0	1	1	1	1	0	0	0
206339	1	3	1	2	1	2	2	2	1	2	0	0	1	1	1	1	0	0	0
2063310	0	2	1	1	1	2	1	2	1	1	0	0	1	1	3	5	1	1	1
2063311	1	5	1	2	1	2	1	1	1	1	0	0	1	1	2	1	1	0	1
2063312	1	2	1	1	1	4	1	1	1	1	0	0	1	1	3	1	1	0	1
2063313	1	2	1	2	1	2	2	1	3	1	0	0	1	1	3	1	1	0	1
2063314	0	4	1	2	1	2	1	1	4	2	0	0	1	1	1	1	0	0	0
2063315	1	4	1	2	1	2	2	1	3	1	0	0	1	1	1	1	0	0	0
2063316	0	1	1	2	1	2	1	2	3	1	0	0	1	1	2	1	1	0	1
2063317	0	2	1	1	1	2	1	2	3	1	0	0	1	1	1	1	0	0	0
2063318	0	4	1	1	1	2	1	2	3	1	0	0	1	1	1	1	0	0	0
2063319	1	2	1	1	1	2	1	2	3	1	0	0	1	1	1	1	0	0	0
2063320	1	3	1	2	1	2	2	2	3	1	0	0	1	1	3	1	1	0	1
2063321	0	3	1	4	1	2	1	2	3	2	0	0	1	1	1	1	0	0	0
2063322	0	1	1	2	1	2	1	1	3	1	0	0	1	1	2	1	1	0	1
2063323	1	2	1	4	1	2	2	2	3	3	0	0	1	1	2	1	1	0	1
2063324	1	4	1	2	1	2	1	1	3	3	0	0	1	1	3	1	1	0	1
2063325	0	5	1	2	1	2	1	1	3	3	0	0	1	1	2	1	1	0	1
2063326	1	5	1	1	1	4	1	1	3	4	0	0	1	1	2	1	1	0	1
2063327	0	5	1	2	1	2	1	1	1	1	0	0	1	1	1	1	0	0	0
2063328	1	3	1	1	1	2	1	2	1	1	0	0	1	1	1	1	0	0	0
2063329	0	2	1	1	1	2	2	2	3	1	0	0	1	1	2	1	1	0	1
2063330	0	1	1	2	1	2	1	1	3	1	0	0	1	1	1	1	0	0	0
307341	1	5	3	4	4	5	3	2	4	1	1	1	1	1	1	1	0	0	0
307342	1	5	3	5	4	5	2	1	4	1	1	1	1	1	1	1	0	0	0
307343	0	2	3	3	4	1	1	2	4	2	0	0	1	1	1	1	0	0	0
307344	1	4	3	2	4	2	2	1	4	1	0	0	1	1	1	1	0	0	0
307345	1	4	3	3	4	2	2	2	4	1	0	0	1	1	1	1	0	0	0
307346	1	4	3	5	4	5	2	2	4	1	1	1	1	1	1	1	0	0	0
307347	0	3	3	4	4	1	2	2	4	3	1	1	1	1	1	1	0	0	0
307348	1	2	3	4	4	5	1	2	4	3	0	0	1	1	1	1	0	0	0
307349	1	3	3	3	4	2	2	1	4	3	0	0	1	1	1	1	0	0	0
3073410	0	2	3	3	4	1	1	2	4	3	0	0	1	1	1	1	0	0	0
3073411	1	1	3	3	4	2	2	2	4	3	0	0	1	1	1	1	0	0	0
3073412	0	1	3	5	4	5	3	1	4	3	1	1	1	1	1	1	0	0	0

3073413	0	1	3	2	4	1	1	2	4	3	0	0	1	1	1	1	0	0	0
3073414	1	5	3	3	4	2	2	2	4	3	0	0	0	99	99	99	99	99	99
3073415	1	4	3	2	4	2	1	2	4	3	0	0	1	1	1	1	0	0	0
3073416	1	2	3	3	4	2	1	1	4	3	0	0	1	1	1	1	0	0	0
3073417	1	2	3	3	4	2	1	2	4	3	0	0	1	1	1	1	0	0	0
3073418	1	4	3	2	4	2	1	1	4	3	0	0	0	99	99	99	99	99	99
3073419	1	2	3	3	4	2	1	2	4	3	0	0	1	1	1	1	0	0	0
3073420	1	2	3	2	4	2	1	2	4	3	0	0	1	1	1	1	0	0	0
3073421	1	3	3	2	4	2	2	1	4	3	0	0	1	1	1	1	0	0	0
3073422	1	3	3	2	4	2	2	2	4	3	0	0	1	1	1	1	0	0	0
3073423	1	4	3	4	4	5	2	2	4	3	0	0	1	1	1	4	0	1	1
3073424	1	2	3	5	4	5	3	2	4	3	1	1	1	1	1	1	0	0	0
3073425	1	3	3	2	4	2	1	2	4	3	0	0	1	1	1	1	0	0	0
3073426	1	4	3	2	4	2	1	1	4	3	0	0	1	1	1	1	0	0	0
3073427	1	4	3	2	4	2	1	2	4	3	0	0	1	1	1	1	0	0	0
3073428	1	1	3	5	4	5	3	2	4	3	1	1	1	1	1	1	0	0	0
3073429	1	2	3	4	4	2	2	1	4	3	0	0	1	1	1	1	0	0	0
3073430	1	4	3	4	4	2	1	1	4	3	0	0	0	99	99	99	99	99	99
3073431	1	4	3	2	4	2	1	1	4	3	0	0	1	1	1	1	0	0	0
3073432	1	2	3	5	4	5	2	2	4	3	1	1	1	1	1	1	0	0	0
307351	0	2	3	5	4	5	3	2	4	2	1	1	1	1	1	1	0	0	0
307352	1	2	3	2	4	2	1	2	4	2	0	0	1	1	1	1	0	0	0
307353	1	2	3	2	4	2	1	2	4	2	0	0	1	1	1	1	0	0	0
307354	0	4	3	2	4	1	1	2	4	2	0	0	1	1	1	1	0	0	0
307355	1	3	3	3	4	2	2	2	4	2	0	0	1	1	1	1	0	0	0
307356	0	2	3	2	4	1	1	2	4	2	0	0	1	1	1	1	0	0	0
307357	0	5	3	2	4	1	1	1	4	2	0	0	1	1	1	1	0	0	0
307358	0	4	3	2	4	1	1	1	4	2	0	0	1	1	1	1	0	0	0
307359	0	2	3	3	4	1	1	1	4	2	0	0	1	1	1	1	0	0	0
3073510	1	2	3	3	4	2	1	2	4	2	0	0	1	1	1	1	0	0	0
3073511	1	5	3	2	4	2	2	1	4	2	0	0	1	1	1	1	0	0	0
3073512	0	1	3	4	4	1	2	2	4	2	1	1	1	1	1	1	0	0	0
3073513	1	3	3	2	4	2	1	2	4	2	0	0	1	1	1	1	0	0	0
3073514	1	3	3	3	4	2	1	2	4	2	0	0	1	1	1	1	0	0	0
3073515	0	3	3	2	4	1	1	2	4	2	0	0	1	1	1	1	0	0	0
3073516	0	3	3	2	4	1	1	2	4	2	0	0	1	1	1	1	0	0	0
3073517	0	4	3	2	4	1	1	2	4	2	0	0	1	1	1	1	0	0	0
3073518	0	2	3	3	4	1	1	1	4	2	0	0	1	1	1	1	0	0	0
3073519	0	2	3	3	4	1	1	2	4	2	0	0	1	1	1	1	0	0	0
3073520	1	2	3	2	4	2	1	2	4	2	0	0	1	1	1	1	0	0	0
3073521	0	2	3	5	4	5	2	1	4	1	1	1	1	1	1	1	0	0	0
3073522	1	2	3	5	4	5	2	1	4	1	1	1	1	1	1	1	0	0	0
3073523	0	3	3	3	4	1	2	2	4	2	0	0	0	99	99	99	99	99	99
3073524	1	3	3	5	4	5	3	2	4	2	1	1	1	1	1	1	0	0	0
3073525	1	2	3	5	4	5	3	1	4	1	1	1	1	1	1	1	0	0	0
3073526	1	4	3	4	4	5	3	1	4	2	1	1	1	1	1	1	0	0	0
3073527	1	1	3	5	4	5	2	1	4	1	1	1	1	1	1	1	0	0	0
3073528	1	3	3	4	4	5	3	2	4	2	1	1	1	1	1	1	0	0	0
3073529	1	2	3	5	4	5	3	1	4	1	1	1	1	1	1	1	0	0	0
3073530	1	2	3	2	4	2	2	1	4	1	0	0	1	1	1	1	0	0	0
3073531	1	2	3	3	4	2	2	2	4	1	0	0	1	1	1	1	0	0	0
3073532	1	5	3	4	4	5	2	1	4	1	1	0	1	1	1	1	0	0	0
3073533	1	2	3	5	4	5	2	1	4	1	1	1	1	1	1	1	0	0	0
307361	1	2	3	4	4	2	2	2	3	1	1	1	1	1	1	1	0	0	0
307362	1	2	3	5	4	5	3	1	3	1	1	1	1	1	1	1	0	0	0
307363	1	5	3	5	4	5	2	1	3	1	1	1	1	1	3	1	1	0	1

307364	1	5	3	2	4	2	2	1	3	1	0	0	1	1	1	1	0	0	0
307365	0	3	3	3	4	1	2	2	3	1	0	0	1	1	1	1	0	0	0
307366	1	4	3	4	4	2	2	2	3	1	0	0	1	1	3	1	1	0	1
307367	1	5	3	2	4	2	1	1	3	4	0	0	1	1	1	1	0	0	0
307368	1	4	3	5	2	5	3	2	3	1	1	1	1	1	1	2	0	0	0
307369	0	4	3	5	4	5	3	2	3	1	1	1	1	1	1	1	0	0	0
3073610	0	2	3	2	4	1	2	1	3	1	0	0	1	1	4	1	1	0	1
3073611	0	4	3	2	4	2	2	1	3	1	0	0	1	1	1	1	0	0	0
3073612	1	3	3	5	4	5	3	1	3	2	0	0	1	1	1	1	0	0	0
3073613	0	2	3	4	4	5	2	1	3	2	1	0	1	1	3	1	1	0	1
3073614	1	5	3	2	4	2	1	1	3	2	0	0	1	1	3	1	1	0	1
3073615	0	3	3	4	4	5	3	1	3	2	1	1	1	1	1	2	0	0	0
3073616	1	4	3	2	4	2	1	2	3	2	0	0	1	1	4	1	1	0	1
3073617	1	4	3	5	4	5	3	1	3	2	1	1	1	1	1	1	0	0	0
3073618	1	2	3	4	4	5	3	1	3	2	0	0	1	1	3	1	1	0	1
3073619	1	5	3	2	4	2	2	1	3	2	0	0	1	1	3	1	1	0	1
3073620	1	2	3	2	4	2	2	2	3	2	0	0	1	1	1	1	0	0	0
3073621	1	2	3	4	4	2	2	2	3	2	0	0	1	1	3	1	1	0	1
3073622	0	3	3	3	4	2	2	2	3	4	0	0	1	1	3	1	1	0	1
3073623	1	5	3	4	4	2	2	1	3	4	0	0	1	1	3	1	1	0	1
3073624	1	4	3	3	4	2	1	2	3	4	0	0	1	1	3	1	1	0	1
3073625	0	5	3	2	4	1	2	1	3	4	0	0	1	1	3	1	1	0	1
3073626	1	4	3	4	4	2	2	2	3	4	0	0	1	1	4	1	1	0	1
3073627	0	3	3	2	4	1	1	1	3	4	0	0	1	1	1	4	0	1	1
3073628	1	5	3	2	4	2	2	2	3	4	0	0	1	1	4	1	1	0	1
3073629	1	2	3	3	4	2	2	2	3	4	0	0	1	1	3	1	1	0	1
3073630	1	4	3	3	4	2	1	2	3	2	0	0	1	1	4	1	1	0	1
307371	0	1	3	4	4	1	2	1	1	3	0	0	1	1	1	1	0	0	0
307372	0	2	3	4	4	1	2	1	1	3	0	0	1	1	1	1	0	0	0
307373	0	2	3	2	4	1	2	2	1	2	0	0	1	1	1	1	0	0	0
307374	0	3	3	2	4	1	2	2	1	1	0	0	0	99	99	99	99	99	99
307375	0	2	3	2	4	2	1	2	1	4	0	0	1	1	3	1	1	0	1
307376	0	1	3	4	4	2	1	1	1	4	0	0	1	1	4	1	1	0	1
307377	1	2	3	2	4	2	2	2	1	1	1	1	1	1	1	1	0	0	0
307378	0	5	3	2	4	1	2	1	1	4	0	0	0	99	99	99	99	99	99
307379	1	2	3	2	4	2	2	2	1	1	0	0	1	1	1	1	0	0	0
3073710	0	3	3	3	4	1	2	1	1	1	0	0	1	1	1	1	0	0	0
3073711	1	1	3	2	4	2	1	1	1	4	0	0	0	99	99	99	99	99	99
3073712	1	5	3	1	4	2	1	1	1	4	0	0	0	99	99	99	99	99	99
3073713	1	1	3	2	2	2	1	1	1	3	0	0	1	1	3	1	1	0	1
3073714	1	4	3	4	4	2	2	2	1	3	0	0	1	1	2	1	1	0	1
3073715	0	2	3	3	4	3	3	2	1	2	1	0	1	1	1	1	0	0	0
3073716	1	2	3	2	4	2	2	1	4	3	0	0	1	1	1	1	0	0	0
3073717	0	1	3	3	4	1	1	1	4	4	0	0	1	1	2	1	1	0	1
3073718	0	2	3	3	4	1	2	2	4	4	0	0	0	99	99	99	99	99	99
3073719	1	5	3	2	4	2	1	1	4	4	0	0	0	99	99	99	99	99	99
3073720	0	2	3	3	4	1	1	1	4	4	0	0	1	1	2	1	1	0	1
3073721	1	5	3	2	4	2	2	2	4	3	0	0	1	1	2	1	1	0	1
3073722	0	2	3	2	4	1	1	1	4	4	0	0	1	1	2	2	1	0	1
3073723	0	3	3	2	4	1	2	2	4	4	0	0	1	1	1	1	0	0	0
3073724	0	1	3	2	4	1	2	1	4	2	0	0	1	1	1	1	0	0	0
3073725	0	4	3	3	4	3	2	1	4	3	0	0	1	1	1	1	0	0	0
3073726	1	2	3	2	4	4	2	1	4	3	0	0	0	99	99	99	99	99	99
3073727	1	2	3	2	4	4	2	2	4	3	0	0	1	1	1	1	0	0	0
3073728	0	1	3	2	4	1	1	2	4	2	0	0	0	99	99	99	99	99	99
3073729	0	2	3	3	4	1	2	2	4	3	0	0	0	99	99	99	99	99	99

3073730	0	1	3	2	3	1	1	1	4	2	0	0	0	99	99	99	99	99	99
307381	0	2	3	2	4	2	2	1	4	1	0	0	1	1	1	4	0	1	1
307382	1	4	3	3	4	2	2	2	4	1	0	0	1	1	1	4	0	1	1
307383	0	2	3	3	4	2	2	2	4	1	0	0	1	1	1	4	0	1	1
307384	1	3	3	2	4	2	2	2	4	1	0	0	1	1	1	4	0	1	1
307385	0	1	3	4	4	2	2	2	4	1	0	0	1	1	1	1	0	0	0
307386	1	3	3	2	4	2	2	2	4	1	0	0	1	1	1	4	0	1	1
307387	0	1	3	3	4	2	2	2	4	1	0	0	1	1	1	4	0	1	1
307388	1	3	3	3	4	2	2	1	4	1	0	0	1	1	3	1	1	0	1
307389	1	3	3	4	4	2	2	1	4	1	0	0	1	1	3	1	1	0	1
3073810	0	2	3	4	4	3	2	1	4	1	1	0	1	1	1	1	0	0	0
3073811	0	2	3	5	4	5	2	2	4	1	1	0	1	1	1	1	0	0	0
3073812	1	2	3	3	4	2	2	2	4	1	0	0	1	1	1	1	0	0	0
3073813	0	3	3	2	4	2	2	1	4	1	0	0	1	1	1	4	0	1	1
3073814	1	2	3	5	4	5	2	2	4	1	0	0	1	1	3	1	1	0	1
3073815	0	2	3	5	4	5	3	1	4	1	1	0	1	1	3	1	1	0	1
3073816	1	2	3	2	4	2	1	2	4	3	0	0	1	1	1	1	0	0	0
3073817	0	2	3	5	4	5	3	1	4	1	1	0	1	1	1	1	0	0	0
3073818	1	4	3	3	4	2	2	2	4	2	0	0	1	1	1	1	0	0	0
3073819	0	1	3	4	4	3	3	1	4	1	1	0	1	1	1	1	0	0	0
3073820	1	3	3	4	4	2	2	2	4	2	0	0	1	1	3	1	1	0	1
3073821	0	2	3	5	4	5	2	1	4	4	0	0	1	1	1	1	0	0	0
3073822	1	4	3	2	4	2	2	2	4	3	0	0	1	1	1	1	0	0	0
3073823	0	2	3	3	4	2	2	2	4	2	0	0	1	1	1	1	0	0	0
3073824	1	4	3	2	4	2	2	2	4	3	0	0	1	1	1	1	0	0	0
3073825	0	1	3	4	4	2	2	1	4	3	0	0	1	1	1	1	0	0	0
3073826	0	4	3	2	4	1	2	1	4	3	0	0	1	1	1	1	0	0	0
3073827	1	4	3	2	2	2	2	2	4	3	0	0	1	1	1	1	0	0	0
3073828	1	4	3	4	4	5	3	2	4	2	1	1	1	1	1	1	0	0	0
3073829	0	2	3	2	4	2	2	2	4	4	0	0	1	1	1	4	0	1	1
3073830	1	4	3	2	4	2	2	2	4	3	0	0	1	1	1	1	0	0	0
307391	1	3	3	4	4	5	2	2	1	3	1	1	1	1	1	1	0	0	0
307392	1	1	3	4	4	2	2	2	2	3	0	0	1	1	1	6	0	1	1
307393	1	2	3	2	4	2	2	2	2	3	0	0	1	1	2	1	1	0	1
307394	0	2	3	2	4	1	2	2	2	3	0	0	0	99	99	99	99	99	99
307395	1	1	3	4	4	2	2	1	2	3	0	0	0	99	99	99	99	99	99
307396	0	2	3	2	4	1	2	2	3	2	0	0	0	99	99	99	99	99	99
307397	1	3	3	2	4	2	2	2	3	2	0	0	0	99	99	99	99	99	99
307398	1	4	3	3	4	2	2	1	2	3	0	0	0	99	99	99	99	99	99
307399	0	4	3	3	4	1	1	1	2	2	0	0	1	1	4	1	1	0	1
3073910	1	4	3	2	4	2	2	2	2	2	0	0	1	1	1	1	0	0	0
3073911	1	5	3	3	4	2	1	1	2	2	0	0	1	1	1	1	0	0	0
3073912	0	1	3	5	4	5	2	1	2	1	0	0	1	1	1	1	0	0	0
3073913	1	3	3	5	4	5	2	1	2	1	1	0	1	1	1	1	0	0	0
3073914	0	2	3	2	4	1	1	2	2	2	0	0	0	99	99	99	99	99	99
3073915	0	1	3	3	4	1	2	1	2	1	0	0	0	99	99	99	99	99	99
3073916	0	2	3	2	4	2	1	2	2	2	0	0	0	99	99	99	99	99	99
3073917	0	2	3	2	4	1	1	2	2	1	0	0	0	99	99	99	99	99	99
3073918	1	4	3	2	4	2	1	1	2	2	0	0	0	99	99	99	99	99	99
3073919	1	4	3	2	4	2	1	2	2	1	0	0	0	99	99	99	99	99	99
3073920	0	2	3	2	4	1	2	1	2	2	0	0	0	99	99	99	99	99	99
3073921	1	2	3	2	4	2	1	1	2	2	0	0	1	1	3	1	1	0	1
3073922	1	5	3	2	4	2	1	2	2	2	0	0	1	1	2	1	1	0	1
3073923	1	2	3	2	4	2	1	1	2	2	0	0	0	99	99	99	99	99	99
3073924	0	2	3	2	4	1	2	2	2	2	0	0	0	99	99	99	99	99	99
3073925	1	5	3	2	4	2	1	1	2	2	0	0	0	99	99	99	99	99	99

3073926	0	2	3	2	4	1	1	1	2	2	0	0	1	1	2	1	1	0	1
3073927	0	2	3	2	4	1	2	2	2	2	0	0	1	1	2	6	1	1	1
3073928	0	3	3	2	4	1	2	1	4	2	0	0	0	99	99	99	99	99	99
3073929	0	3	3	2	4	2	1	1	2	2	0	0	0	99	99	99	99	99	99
3073930	1	2	3	4	4	5	2	2	2	3	0	0	0	99	99	99	99	99	99
307401	0	3	3	3	4	2	2	1	2	4	0	0	1	1	3	1	1	0	1
307402	1	3	3	2	4	2	1	1	2	4	0	0	1	1	3	1	1	0	1
307403	0	2	3	2	4	2	1	2	2	4	0	0	1	1	1	4	0	1	1
307404	1	3	3	3	4	2	2	2	2	4	0	0	1	1	1	4	0	1	1
307405	1	3	3	2	4	2	2	2	2	4	0	0	1	1	1	4	0	1	1
307406	1	3	3	3	4	2	2	2	2	4	0	0	1	1	1	4	0	1	1
307407	0	2	3	2	4	2	2	2	2	4	0	0	1	1	3	1	1	0	1
307408	0	1	3	4	4	2	2	2	2	4	0	0	1	1	3	1	1	0	1
307409	0	3	3	3	4	2	2	2	2	4	0	0	1	1	1	4	0	1	1
3074010	1	1	3	5	4	5	2	1	2	4	0	0	1	1	1	1	0	0	0
3074011	1	3	3	2	4	2	1	1	2	1	0	0	1	1	3	1	1	0	1
3074012	1	2	3	5	4	5	3	1	2	1	1	0	1	1	3	1	1	0	1
3074013	0	1	3	4	4	2	2	1	2	1	0	0	1	1	3	1	1	0	1
3074014	0	2	3	3	4	2	2	2	2	1	0	0	1	1	1	4	0	1	1
3074015	0	2	3	4	4	2	2	1	2	1	0	0	1	1	1	4	0	1	1
3074016	1	2	3	5	4	5	3	2	2	1	1	1	1	1	1	1	0	0	0
3074017	0	2	3	2	4	2	1	2	2	1	0	0	1	1	1	4	0	1	1
3074018	1	2	3	2	4	2	1	1	2	3	0	0	1	1	1	4	0	1	1
3074019	1	1	3	3	4	2	1	2	2	3	0	0	1	1	1	1	0	0	0
3074020	1	1	3	2	4	2	1	1	2	1	0	0	1	1	1	4	0	1	1
3074021	0	2	3	3	4	2	2	2	2	4	1	0	1	1	1	4	0	1	1
3074022	0	2	3	2	4	2	1	2	2	3	0	0	1	1	1	1	0	0	0
3074023	0	2	3	3	4	2	2	2	2	3	0	0	1	1	1	1	0	0	0
3074024	1	5	3	2	4	2	2	2	2	3	0	0	1	1	1	1	0	0	0
3074025	0	3	3	2	4	2	1	2	2	3	0	0	1	1	1	1	0	0	0
3074026	0	2	3	4	4	2	2	1	2	3	0	0	1	1	3	1	1	0	1
3074027	1	1	3	3	4	2	2	1	2	3	0	0	1	1	1	1	0	0	0
3074028	0	3	3	2	4	2	2	2	2	3	0	0	1	1	1	4	0	1	1
3074029	1	2	3	3	4	2	2	2	2	3	0	0	1	1	1	4	0	1	1
3074030	1	3	3	3	4	2	1	1	2	1	0	0	1	1	1	4	0	1	1
3074031	1	2	3	2	4	2	2	2	2	2	0	0	1	1	1	1	0	0	0
3074032	0	1	3	4	4	2	2	1	2	1	0	0	1	1	1	1	0	0	0
3074033	0	1	3	5	4	5	2	1	2	1	0	0	1	1	1	1	0	0	0
3074034	1	4	3	4	4	2	1	1	2	1	0	0	1	1	3	1	1	0	1
3074035	0	2	3	5	4	5	2	1	2	1	1	0	1	1	3	1	1	0	1
307411	0	2	3	2	2	1	2	2	3	2	0	0	1	1	1	1	0	0	0
307412	0	4	3	2	4	1	2	1	3	2	0	0	1	1	1	1	0	0	0
307413	0	3	3	2	4	1	2	2	3	3	0	0	1	1	1	1	0	0	0
307414	0	1	3	4	4	1	1	2	3	3	0	0	1	1	1	1	0	0	0
307415	0	2	3	2	4	1	2	2	2	2	0	0	1	1	1	1	0	0	0
307416	1	3	3	2	4	2	2	2	2	2	0	0	1	1	2	1	1	0	1
307417	0	1	3	2	4	1	2	2	3	2	0	0	1	1	1	1	0	0	0
307418	0	2	3	4	4	3	2	1	3	3	0	0	1	1	2	1	1	0	1
307419	0	2	3	5	2	5	2	2	3	3	0	0	1	1	2	1	1	0	1
3074110	0	2	3	2	4	1	2	2	3	2	0	0	1	1	2	1	1	0	1
3074111	1	1	3	3	4	2	2	2	3	2	0	0	1	1	2	1	1	0	1
3074112	0	2	3	4	4	1	1	2	3	2	0	0	1	1	2	1	1	0	1
3074113	0	2	3	2	4	1	2	2	3	2	0	0	1	1	1	1	0	0	0
3074114	1	2	3	3	4	2	2	1	3	2	0	0	1	1	1	1	0	0	0
3074115	1	3	3	3	4	4	2	1	3	2	0	0	1	1	1	1	0	0	0
3074116	1	3	3	2	4	3	3	2	3	2	0	0	1	1	1	1	0	0	0

3074117	1	3	3	2	4	2	2	2	3	2	0	0	1	1	1	1	0	0	0
3074118	0	3	3	3	4	1	2	2	3	2	0	0	1	1	1	1	0	0	0
3074119	0	4	3	5	4	5	2	1	3	2	0	0	1	1	1	1	0	0	0
3074120	1	5	3	5	4	5	3	2	3	1	1	1	1	1	1	1	0	0	0
3074121	1	4	3	3	4	2	2	1	3	3	0	0	1	1	1	1	0	0	0
3074122	1	3	3	3	4	5	2	1	3	1	0	0	1	1	1	1	0	0	0
3074123	0	4	3	3	4	1	2	1	3	1	0	0	1	1	1	1	0	0	0
3074124	0	5	3	2	4	1	1	1	3	1	0	0	1	1	1	1	0	0	0
3074125	1	5	3	2	4	2	2	2	3	1	0	0	1	1	1	1	0	0	0
3074126	0	2	3	5	4	5	2	1	3	1	0	0	1	1	1	1	0	0	0
3074127	0	5	3	2	4	1	2	2	3	1	0	0	1	1	1	1	0	0	0
3074128	0	5	3	2	4	1	2	2	3	3	0	0	1	1	1	1	0	0	0
3074129	1	4	3	5	4	5	2	2	3	3	1	0	1	1	4	1	1	0	1
3074130	0	2	3	3	4	1	3	2	3	2	1	0	1	1	1	1	0	0	0
3074131	1	5	3	2	4	2	2	2	3	2	0	0	1	1	2	1	1	0	1
3074132	0	2	3	2	4	1	2	2	4	4	0	0	0	99	99	99	99	99	99
3074133	1	4	3	2	4	2	2	1	4	4	0	0	0	99	99	99	99	99	99
3074134	0	2	3	5	4	5	3	1	4	3	1	1	1	1	1	1	0	0	0
3074135	1	5	3	2	4	2	2	1	4	3	0	0	1	1	2	1	1	0	1
3074136	1	5	3	2	4	2	2	1	4	4	0	0	1	1	1	1	0	0	0
3074137	1	5	3	2	4	2	2	2	4	3	0	0	0	99	99	99	99	99	99
3074138	0	3	3	3	4	1	2	1	4	4	0	0	1	1	2	1	1	0	1
3074139	1	3	3	2	4	4	2	1	4	3	0	0	0	99	99	99	99	99	99
3074140	1	5	3	2	4	2	2	2	4	4	0	0	0	99	99	99	99	99	99
308421	0	4	3	2	4	2	1	1	4	4	0	0	0	99	99	99	99	99	99
308422	1	4	3	2	4	2	2	2	4	4	0	0	1	1	4	1	1	0	1
308423	0	3	3	3	4	2	3	2	2	4	0	0	1	1	4	1	1	0	1
308424	0	2	3	2	4	2	1	1	2	4	0	0	1	1	4	1	1	0	1
308425	0	3	3	2	4	2	3	2	2	4	0	0	1	1	1	1	0	0	0
308426	0	2	3	2	4	2	3	2	2	4	0	0	1	1	1	1	0	0	0
308427	1	2	3	2	4	2	1	2	4	4	0	0	0	99	99	99	99	99	99
308428	1	4	3	2	4	2	2	2	4	4	0	0	1	1	2	1	1	0	1
308429	0	1	3	4	4	2	2	2	4	4	0	0	1	1	3	1	1	0	1
3084210	0	2	3	2	4	2	2	1	2	4	0	0	0	99	99	99	99	99	99
3084211	1	3	3	2	4	2	1	2	2	3	0	0	0	99	99	99	99	99	99
3084212	0	3	3	1	4	2	2	1	2	1	0	0	0	99	99	99	99	99	99
3084213	0	2	3	2	4	2	2	2	4	4	0	0	0	99	99	99	99	99	99
3084214	0	2	3	2	4	2	2	1	4	4	0	0	0	99	99	99	99	99	99
3084215	1	2	3	2	4	2	1	1	2	4	0	0	0	99	99	99	99	99	99
3084216	1	3	3	2	4	2	2	2	2	4	0	0	0	99	99	99	99	99	99
3084217	1	2	3	2	4	2	2	1	2	1	0	0	0	99	99	99	99	99	99
3084218	1	2	3	2	4	2	2	1	4	1	0	0	1	1	3	1	1	0	1
3084219	1	3	3	3	4	2	2	1	4	1	0	0	1	1	1	1	0	0	0
3084220	1	4	3	4	4	5	3	2	4	1	1	1	0	99	99	99	99	99	99
3084221	0	2	3	2	4	3	2	2	4	1	1	1	1	1	1	1	0	0	0
3084222	0	4	3	3	4	1	2	2	4	1	0	0	0	99	99	99	99	99	99
3084223	0	4	3	2	4	1	1	1	4	1	0	0	1	1	1	1	0	0	0
3084224	0	2	3	3	4	1	2	2	4	1	0	0	0	99	99	99	99	99	99
3084225	1	3	3	2	4	2	2	2	4	2	0	0	0	99	99	99	99	99	99
3084226	0	2	3	4	4	1	3	2	4	3	0	0	1	1	1	1	0	0	0
3084227	1	4	3	2	4	2	2	2	4	3	0	0	0	99	99	99	99	99	99
3084228	1	5	3	1	4	2	2	1	4	2	0	0	0	99	99	99	99	99	99
3084229	0	1	3	3	4	1	3	2	4	2	0	0	1	1	1	1	0	0	0
3084230	1	4	3	2	4	5	2	2	4	1	1	1	0	99	99	99	99	99	99
308431	0	3	3	2	4	1	2	2	2	4	0	0	1	1	1	1	0	0	0
308432	1	5	3	4	4	5	2	1	2	4	0	0	1	1	2	1	1	0	1

308433	0	2	3	2	4	2	1	1	2	4	0	0	1	1	2	1	1	0	1
308434	0	4	3	2	4	2	2	1	2	4	0	0	0	99	99	99	99	99	99
308435	0	2	3	4	4	2	1	1	2	4	0	0	1	1	1	1	0	0	0
308436	1	1	3	4	4	5	2	1	2	4	0	0	1	1	2	1	1	0	1
308437	0	1	3	5	4	5	2	2	2	4	1	0	0	99	99	99	99	99	99
308438	1	2	3	2	4	3	2	1	2	3	0	0	0	99	99	99	99	99	99
308439	0	2	3	2	4	2	2	2	2	4	0	0	0	99	99	99	99	99	99
3084310	1	3	3	3	4	2	1	2	2	3	0	0	1	1	1	1	0	0	0
3084311	0	2	3	2	4	2	2	2	2	4	0	0	1	1	3	1	1	0	1
3084312	0	2	3	2	2	1	2	2	4	1	0	0	0	99	99	99	99	99	99
3084313	1	5	3	2	4	2	2	1	4	2	0	0	0	99	99	99	99	99	99
3084314	0	1	3	4	4	1	2	2	4	4	0	0	0	99	99	99	99	99	99
3084315	1	4	3	4	4	3	3	2	4	2	0	0	0	99	99	99	99	99	99
3084316	1	1	3	2	4	2	3	1	4	4	0	0	1	1	1	6	0	1	1
3084317	1	1	3	4	2	2	2	2	4	3	0	0	1	1	1	6	0	1	1
3084318	0	1	3	2	4	1	2	1	4	4	0	0	0	99	99	99	99	99	99
3084319	0	2	3	2	4	1	2	2	4	4	0	0	0	99	99	99	99	99	99
3084320	1	2	3	3	4	2	3	2	4	4	0	0	0	99	99	99	99	99	99
3084321	1	5	3	2	4	2	2	2	4	2	0	0	1	1	4	1	1	0	1
3084322	0	3	3	2	4	3	3	2	4	2	0	0	0	99	99	99	99	99	99
3084323	0	3	3	2	4	2	2	2	4	3	0	0	0	99	99	99	99	99	99
3084324	1	1	3	2	4	2	3	1	4	4	0	0	0	99	99	99	99	99	99
3084325	0	3	3	4	4	1	3	2	4	1	0	0	0	99	99	99	99	99	99
3084326	0	1	3	2	4	2	1	1	4	4	0	0	0	99	99	99	99	99	99
3084327	0	1	3	2	4	2	1	1	4	4	0	0	0	99	99	99	99	99	99
3084328	0	1	3	5	4	5	2	2	4	4	0	0	0	99	99	99	99	99	99
3084329	1	5	3	5	4	5	3	2	4	1	1	1	1	1	2	1	1	0	1
3084330	0	2	3	2	4	2	2	1	4	4	0	0	0	99	99	99	99	99	99
3084331	1	4	3	4	4	2	2	1	4	4	0	0	1	1	4	1	1	0	1
3084332	1	2	3	5	4	5	2	1	4	3	1	1	1	1	1	6	0	1	1
3084333	0	2	3	5	4	5	3	1	4	4	1	1	0	99	99	99	99	99	99
3084334	1	4	3	5	4	5	3	2	4	4	1	1	0	99	99	99	99	99	99
3084335	1	4	3	5	4	5	3	1	4	3	0	0	1	1	1	1	0	0	0
3084336	1	4	3	5	3	5	3	2	4	4	1	1	1	1	1	1	0	0	0
3084337	0	4	3	2	4	1	3	1	4	4	0	0	1	1	1	1	0	0	0
3084338	1	3	3	4	4	3	3	2	4	3	1	0	0	99	99	99	99	99	99
3084339	1	4	3	3	4	2	3	1	4	4	0	0	1	1	3	1	1	0	1
308441	0	2	3	3	4	2	2	1	2	4	0	0	1	1	2	1	1	0	1
308442	0	2	3	2	4	2	2	2	2	4	0	0	0	99	99	99	99	99	99
308443	0	2	3	2	4	2	2	2	2	1	0	0	0	99	99	99	99	99	99
308444	0	5	3	2	4	2	2	1	2	1	0	0	1	1	1	1	0	0	0
308445	0	5	3	2	4	2	2	1	2	1	0	0	1	1	3	1	1	0	1
308446	1	4	3	2	4	2	2	2	2	1	0	0	1	1	3	1	1	0	1
308447	1	5	3	2	4	2	2	1	2	3	1	1	0	99	99	99	99	99	99
308448	1	5	3	2	4	2	2	2	2	4	0	0	0	99	99	99	99	99	99
308449	0	1	3	2	4	2	1	1	2	4	0	0	1	1	3	1	1	0	1
3084410	1	3	3	3	4	2	2	1	2	4	0	0	0	99	99	99	99	99	99
3084411	1	5	3	1	4	2	2	2	2	2	0	0	0	99	99	99	99	99	99
3084412	1	3	3	4	4	3	3	1	2	1	1	0	1	1	4	6	1	1	1
3084413	1	5	3	2	4	2	2	1	2	1	0	0	1	1	4	1	1	0	1
3084414	1	3	3	4	4	5	3	2	2	1	0	0	1	1	1	1	0	0	0
3084415	1	1	3	2	4	4	3	2	2	1	0	0	1	1	3	1	1	0	1
3084416	0	3	3	2	4	2	2	1	2	1	0	0	0	99	99	99	99	99	99
3084417	0	1	3	5	4	5	3	2	2	3	0	0	1	1	3	1	1	0	1
3084418	1	2	3	3	4	2	3	1	2	3	1	1	0	99	99	99	99	99	99
3084419	1	1	3	4	4	2	2	1	2	3	0	0	1	1	3	1	1	0	1

3084420	1	5	3	2	4	2	1	2	2	1	0	0	0	99	99	99	99	99	99
3084421	0	2	3	3	4	1	3	1	2	4	0	0	0	99	99	99	99	99	99
3084422	0	2	3	2	4	2	2	2	2	4	0	0	0	99	99	99	99	99	99
3084423	1	2	3	5	4	5	3	1	2	4	0	0	1	1	1	1	0	0	0
3084424	1	1	3	5	4	5	3	1	2	4	0	0	1	1	1	6	0	1	1
3084425	1	2	3	4	4	5	3	2	2	4	1	0	0	99	99	99	99	99	99
3084426	0	1	3	5	4	5	3	1	2	3	1	0	1	1	1	1	0	0	0
308451	0	4	3	1	4	2	2	2	3	2	0	0	1	1	1	1	0	0	0
308452	1	2	3	2	4	2	1	2	3	2	0	0	1	1	1	4	0	1	1
308453	0	3	3	2	4	2	2	1	3	2	0	0	0	99	99	99	99	99	99
308454	0	2	3	2	4	2	1	2	3	3	0	0	0	99	99	99	99	99	99
308455	0	4	3	1	4	2	1	1	3	2	0	0	1	1	1	6	0	1	1
308456	1	2	3	2	4	2	2	2	1	2	0	0	1	1	1	1	0	0	0
308457	1	2	3	2	4	2	1	2	3	3	0	0	1	1	1	4	0	1	1
308458	0	3	3	2	4	2	2	1	3	3	0	0	0	99	99	99	99	99	99
308459	0	2	3	1	4	2	1	1	3	2	0	0	1	1	1	4	0	1	1
3084510	0	2	3	2	4	2	2	1	3	2	0	0	1	1	1	4	0	1	1
3084511	1	4	3	1	4	2	1	2	3	2	0	0	1	1	1	4	0	1	1
3084512	0	2	3	2	4	2	1	2	3	2	0	0	1	1	1	4	0	1	1
3084513	0	1	3	2	4	2	1	1	3	2	0	0	1	1	1	4	0	1	1
3084514	0	1	3	2	4	2	1	1	3	2	0	0	1	1	1	4	0	1	1
3084515	1	4	3	1	4	2	1	1	3	2	0	0	1	1	1	4	0	1	1
3084516	1	4	3	2	4	2	1	2	3	2	0	0	1	1	1	4	0	1	1
3084517	0	4	3	2	4	2	1	2	3	2	0	0	1	1	3	4	1	1	1
3084518	1	3	3	2	4	2	1	2	3	2	0	0	1	1	1	4	0	1	1
3084519	0	4	3	1	4	2	1	1	3	2	0	0	1	1	1	4	0	1	1
3084520	0	5	3	2	4	2	1	1	3	2	0	0	1	1	1	4	0	1	1
3084521	0	4	3	2	4	2	1	2	3	2	0	0	1	1	1	4	0	1	1
3084522	0	2	3	2	4	2	2	2	3	2	0	0	1	1	1	6	0	1	1
3084523	0	1	3	2	4	2	1	1	3	2	0	0	1	1	1	6	0	1	1
3084524	1	4	3	2	4	2	1	2	3	2	0	0	1	1	1	1	0	0	0
3084525	1	5	3	2	4	2	1	1	3	2	0	0	1	1	1	4	0	1	1
308461	0	4	3	3	4	2	3	2	3	2	1	1	1	1	1	6	0	1	1
308462	1	3	3	1	4	2	1	1	3	2	0	0	1	1	1	1	0	0	0
308463	1	2	3	2	4	2	2	1	3	2	0	0	1	1	1	4	0	1	1
308464	1	4	3	1	4	2	1	2	3	2	0	0	1	1	1	4	0	1	1
308465	0	5	3	2	4	2	1	1	3	2	0	0	1	1	1	4	0	1	1
308466	1	3	3	2	4	2	1	1	3	2	0	0	1	1	2	1	1	0	1
308467	0	4	3	2	4	2	2	1	3	2	0	0	1	1	1	6	0	1	1
308468	0	3	3	2	4	2	2	2	3	2	0	0	1	1	1	1	0	0	0
308469	0	5	3	2	4	2	1	1	3	2	0	0	1	1	1	4	0	1	1
3084610	1	2	3	2	4	2	1	1	3	2	0	0	1	1	1	1	0	0	0
3084611	1	2	3	2	4	2	1	2	3	2	0	0	1	1	1	4	0	1	1
3084612	0	3	3	2	4	2	2	1	3	2	0	0	1	1	1	6	0	1	1
3084613	0	3	3	4	4	2	2	1	3	2	0	0	1	1	1	1	0	0	0
3084614	1	5	3	2	4	2	1	1	3	2	0	0	1	1	1	4	0	1	1
3084615	1	5	3	2	4	2	2	1	3	2	0	0	1	1	1	4	0	1	1
3084616	1	4	3	4	4	2	1	1	3	2	0	0	1	1	1	4	0	1	1
3084617	0	4	3	2	4	2	1	2	3	2	0	0	1	1	1	4	0	1	1
3084618	1	4	3	2	4	2	1	2	3	2	0	0	1	1	1	4	0	1	1
3084619	1	4	3	2	4	2	1	2	3	2	0	0	1	1	1	4	0	1	1
3084620	1	3	3	2	4	2	1	1	3	2	0	0	1	1	1	4	0	1	1
3084621	0	5	3	1	4	2	1	2	3	2	0	0	1	1	1	4	0	1	1
3084622	1	3	3	2	4	2	1	1	3	2	0	0	1	1	1	4	0	1	1
3084623	1	4	3	2	4	2	1	1	3	2	0	0	1	1	1	4	0	1	1
3084624	1	3	3	2	4	2	1	2	3	2	0	0	1	1	1	4	0	1	1

3084625	0	4	3	2	4	2	1	1	3	2	0	0	1	1	1	4	0	1	1
309471	1	4	3	4	4	2	2	2	4	2	1	1	1	1	1	1	0	0	0
309472	0	3	3	3	4	2	1	1	4	1	0	0	1	1	2	1	1	0	1
309473	1	3	3	2	4	2	1	2	4	2	0	0	1	1	1	1	0	0	0
309474	1	4	3	2	4	2	1	2	4	1	1	0	1	1	1	1	0	0	0
309475	0	2	3	2	4	2	1	1	4	1	1	1	1	1	1	1	0	0	0
309476	0	3	3	4	4	1	3	2	4	4	1	1	1	1	1	1	0	0	0
309477	1	2	3	2	4	2	2	2	4	1	0	0	1	1	2	1	1	0	1
309478	1	4	3	4	4	2	1	2	4	1	1	0	1	1	1	1	0	0	0
309479	1	5	3	2	4	2	1	1	4	4	1	0	1	1	2	4	1	1	1
3094710	1	2	3	2	4	2	3	1	4	4	0	0	1	1	2	1	1	0	1
3094711	0	2	3	2	4	2	1	1	4	4	0	0	1	1	2	1	1	0	1
3094712	0	2	3	5	4	5	2	2	4	3	0	0	1	1	1	1	0	0	0
3094713	1	1	3	2	4	2	2	2	4	4	0	0	1	1	2	4	1	1	1
3094714	1	1	3	2	4	2	1	1	4	4	0	0	1	1	3	1	1	0	1
3094715	1	4	3	2	4	2	1	1	4	4	0	0	1	1	1	4	0	1	1
3094716	1	2	3	2	4	2	2	2	4	4	0	0	1	1	3	1	1	0	1
3094717	1	2	3	5	4	5	3	1	4	1	1	0	1	1	1	1	0	0	0
3094718	1	2	3	2	4	2	1	2	4	2	0	0	1	1	3	1	1	0	1
3094719	0	2	3	3	4	2	2	2	4	2	0	0	1	1	2	1	1	0	1
3094720	0	4	3	3	4	2	1	1	4	2	0	0	1	1	2	1	1	0	1
3094721	1	3	3	2	4	2	1	2	4	2	0	0	1	1	2	1	1	0	1
3094722	0	3	3	2	4	2	1	2	4	2	0	0	1	1	2	1	1	0	1
3094723	1	3	3	3	4	2	1	2	4	2	0	0	1	1	2	1	1	0	1
3094724	0	1	3	5	4	5	2	2	4	1	0	0	1	1	2	1	1	0	1
3094725	1	5	3	2	4	2	1	1	4	1	0	0	1	1	2	1	1	0	1
3094726	0	4	3	2	4	2	1	1	4	1	0	0	1	1	2	1	1	0	1
3094727	1	3	3	3	4	2	1	2	4	1	0	0	1	1	2	1	1	0	1
3094728	0	1	3	4	4	2	2	1	4	1	0	0	1	1	2	1	1	0	1
3094729	0	3	3	4	4	2	1	1	4	2	0	0	1	1	2	1	1	0	1
3094730	1	3	3	3	4	2	1	2	4	2	0	0	1	1	2	1	1	0	1
3094731	0	3	3	2	4	2	1	2	4	2	0	0	1	1	2	1	1	0	1
3094732	1	2	3	2	4	2	1	1	4	2	0	0	1	1	2	1	1	0	1
3094733	0	2	3	2	4	2	2	2	4	1	0	0	1	1	2	1	1	0	1
3094734	0	2	3	4	4	2	3	2	4	1	0	0	1	1	2	1	1	0	1
3094735	0	3	3	4	4	2	1	1	4	2	0	0	1	1	2	1	1	0	1
3094736	0	3	3	4	4	2	1	1	4	1	0	0	1	1	2	1	1	0	1
3094737	0	4	3	3	4	2	2	1	4	4	0	0	1	1	3	1	1	0	1
3094738	0	3	3	3	4	2	1	1	4	2	0	0	1	1	2	1	1	0	1
3094739	0	3	3	2	4	2	1	2	4	2	0	0	1	1	4	1	1	0	1
3094740	0	3	3	4	4	2	2	1	4	2	1	0	1	1	1	1	0	0	0
309481	1	2	3	3	4	2	1	1	2	1	0	0	1	1	2	1	1	0	1
309482	1	2	3	3	4	2	1	1	2	4	0	0	1	1	2	1	1	0	1
309483	0	2	3	5	4	5	1	2	2	1	0	0	1	1	3	1	1	0	1
309484	0	5	3	2	4	2	1	2	2	1	0	0	1	1	3	1	1	0	1
309485	0	2	3	4	4	2	2	2	2	1	0	0	1	1	2	1	1	0	1
309486	0	3	3	2	4	2	2	2	2	1	0	0	1	1	1	1	0	0	0
309487	0	3	3	2	4	2	1	2	2	1	0	0	1	1	3	1	1	0	1
309488	0	4	3	2	4	2	1	2	2	1	0	0	1	1	1	1	0	0	0
309489	0	3	3	2	4	2	2	1	2	1	0	0	1	1	2	1	1	0	1
3094810	0	3	3	2	4	2	1	1	2	2	0	0	1	1	3	1	1	0	1
3094811	0	4	3	2	4	2	1	2	2	2	0	0	1	1	3	1	1	0	1
3094812	1	5	3	2	4	2	1	2	2	2	0	0	1	1	2	1	1	0	1
3094813	1	3	3	2	4	2	1	1	2	1	0	0	1	1	1	1	0	0	0
3094814	1	2	3	2	4	2	2	1	2	1	0	0	1	1	3	1	1	0	1
3094815	1	2	3	4	4	2	1	2	2	1	0	0	1	1	2	1	1	0	1

3094816	0	2	3	2	4	2	1	2	2	2	0	0	1	1	2	1	1	0	1
3094817	1	2	3	2	4	2	1	1	2	2	0	0	1	1	3	1	1	0	1
3094818	1	1	3	2	4	2	1	1	2	2	0	0	1	1	3	1	1	0	1
3094819	1	3	3	2	4	2	1	2	2	2	0	0	1	1	3	1	1	0	1
3094820	0	2	3	2	4	2	1	1	2	2	0	0	1	1	2	1	1	0	1
3094821	1	3	3	4	4	2	1	2	2	2	0	0	1	1	3	1	1	0	1
3094822	1	5	3	2	4	2	1	2	2	1	0	0	1	1	2	1	1	0	1
3094823	0	4	3	2	4	2	1	1	2	1	0	0	1	1	2	1	1	0	1
3094824	1	5	3	2	4	2	1	2	2	2	0	0	1	1	2	1	1	0	1
3094825	1	4	3	5	4	2	2	2	2	2	0	0	1	1	2	1	1	0	1
309491	1	4	3	2	4	2	1	2	2	1	0	0	1	1	1	4	0	1	1
309492	0	2	3	2	4	2	1	1	2	4	0	0	1	1	4	1	1	0	1
309493	0	4	3	2	4	2	1	2	2	4	0	0	1	1	2	1	1	0	1
309494	0	5	3	2	4	2	1	1	2	3	0	0	1	1	3	1	1	0	1
309495	0	2	3	2	4	2	1	1	2	1	0	0	1	1	2	1	1	0	1
309496	1	3	3	5	4	5	2	1	2	1	1	1	1	1	1	1	0	0	0
309497	1	4	3	2	4	2	1	1	2	2	0	0	1	1	2	1	1	0	1
309498	0	1	3	2	4	2	1	1	2	4	0	0	1	1	3	1	1	0	1
309499	0	3	3	2	4	2	1	2	2	4	0	0	1	1	3	1	1	0	1
3094910	1	3	3	3	4	2	2	2	2	4	0	0	1	1	4	1	1	0	1
3094911	0	3	3	2	4	2	1	2	2	4	0	0	1	1	3	1	1	0	1
3094912	0	2	3	5	4	5	2	1	2	4	0	0	1	1	4	1	1	0	1
3094913	1	2	3	2	4	2	1	1	3	4	0	0	1	1	2	1	1	0	1
3094914	1	2	3	3	4	2	1	2	2	1	0	0	1	1	1	1	0	0	0
3094915	1	3	3	2	4	2	1	1	2	1	0	0	1	1	2	1	1	0	1
3094916	1	4	3	2	4	2	1	1	2	1	0	0	1	1	1	1	0	0	0
3094917	1	2	3	2	4	2	1	1	2	1	0	0	1	1	1	1	0	0	0
3094918	0	2	3	4	4	2	1	2	2	4	0	0	1	1	1	4	0	1	1
3094919	0	2	3	2	4	2	1	2	2	4	0	0	1	1	3	1	1	0	1
3094920	0	3	3	2	4	2	2	1	2	4	1	0	1	1	3	1	1	0	1
3094921	1	3	3	2	4	2	1	2	2	4	0	0	1	1	1	4	0	1	1
3094922	0	3	3	2	4	2	1	2	2	4	0	0	1	1	3	1	1	0	1
3094923	0	2	3	2	4	2	1	1	2	1	0	0	1	1	1	1	0	0	0
3094924	0	1	3	5	4	5	3	1	2	4	0	0	1	1	3	1	1	0	1
3094925	0	2	3	5	4	5	1	2	2	4	0	0	1	1	3	1	1	0	1
3094926	1	4	3	4	4	2	2	1	2	1	0	0	1	1	1	1	0	0	0
3094927	1	4	3	2	4	2	2	1	2	1	0	0	1	1	1	4	0	1	1
3094928	1	2	3	5	4	5	2	1	2	1	1	1	1	1	1	1	0	0	0
3094929	1	1	3	2	4	2	1	1	2	1	0	0	1	1	2	1	1	0	1
3094930	1	3	3	2	4	2	1	2	2	1	0	0	1	1	2	1	1	0	1

Variables explanation

RID Respondent ID

G Gender of participants

- 0 Female
- 1 Male

AG Age group of participants

- 1 < 30
- 2 30 – 39
- 3 40 – 49
- 4 50 – 59
- 5 ≥ 60

D	District in ENTP	
	1	East Sumba
	2	Belu
	3	East Manggarai
Edu	Education level of participants	
	1	No education
	2	Primary school
	3	Junior high school
	4	Senior high school'
	5	Diploma or above
Eth	Ethnicity in ENTP	
	1	Sumba
	2	Others
	3	Timor
	4	Manggarai
Job	Occupation of participants	
	1	Housewife
	2	Farmer
	3	Enterpreneur/Salesman
	4	Other
	5	Govt. or non-govt. employment
SES	Social economic status of participants	
	1	Low
	2	Average
	3	High
FZ	Family size	
	1	≤ 4
	2	> 4
HF	The nearest health facilities	
	1	Village maternity posts
	2	Village health Post
	3	Subsidiary Public Health centres
	4	Public Health centres
DHF	Distance to the nearest health facilities	
	1	< 1 Km
	2	1 – 2 Km
	3	2 – 3 Km
	4	≥ 3 Km
Inc1	Category of Household Income based on Provincial Minimum Wages (PMW)	
	0	Less than PMW
	1	At least PMW
Inc2	Category of Household Income based on Indonesia Poverty Line (IPL)	
	0	Less than IPL
	1	At least IPL
Q1	Hearing malaria term	
	0	No
	1	Yes
Q2	Seeking treatment if participants or their family members have symptoms of malaria	
	0	No
	1	Yes
	99	Not applicable

Q3	How fast finding treatment if malaria	
	1	One day (Within 24 hours)
	2	2 days
	3	3 days
	4	4 days or more
	5	I did not go for treatment
	99	Not applicable
Q4	Place for seeking treatment if participants or their family members have symptoms of malaria	
	1	Public health facilities
	2	Private health facilities
	4	Traditional healer
	5	Self-treatment
	6	Buying medicine at kiosk
	7	Self-treatment with consuming papaya leaves
	99	Not applicable
O1	Seeking treatment after 24 hours	
	0	No
	1	Yes
	99	Not applicable
O2	Seeking treatment at non-health facilities	
	0	No
	1	Yes
	99	Not applicable
O3	Poor understanding of appropriate malaria treatment-seeking behaviour (AMTSB) (Seeking treatment after 24 hours or at non-health facilities)	
	0	No
	1	Yes
	99	Not applicable
