# Supplementary material for: Malaria treatment-seeking behaviour and its associated factors: A cross-sectional study in rural East Nusa Tenggara Province, Indonesia
Source: PLoS One. 2022 Feb 4;17(2):e0263178. doi: 10.1371/journal.pone.0263178 (PMC8815915; doi:10.1371/journal.pone.0263178)
Supplement: S1 Questionnaire — (PDF) [file pone.0263178.s003.pdf]

QUESTIONNAIRE

Questionnaire

Date of interview : ________/_________/ 2019

Study ID : 20191428-1490

Name of interviewer : ________________________

Mobile number : ________________________

Interviewer initial : ________________________

Participant Info

Name : _________________________

Mobile Number : _________________________

Alternate Number : _________________________

Address : _________________________

Hamlet /Village : ____________/____________

Category of Village : 1 = Easy access 2 = Moderate access 3 = Difficult access

The availability of village malaria post : 1 = Yes 2 = No

Sub-District/District : ____________/____________

Geographical conditions : 1 = Coastal area 2 = Rice fields 3 = Hills 4 = Forest 5 = Swamps

SECTION 1: DEMOGRAPHIC CHARACTERISTICS		
**Questions**	**Coding Categories**	**Answer Code**
1.1 How old are you?	……. years (age in current year)	
1.2 What is your gender?	1 = Male 2 = Female	
1.3 What is your highest level of education?	1 = No education<br>2 = Primary school<br>3 = Junior High school<br>4 = Senior High School<br>5 = Diploma<br>6 = Bachelor or above	
1.4 What is your main occupation?	1= Farmer<br>2 = Housewife<br>3= Fisherman<br>4 = Labour<br>5 = Trader/Merchandiser<br>6 = Student<br>7 = Unemployed<br>8 = unable to work<br>9 = Govt. or non-govt. employment (Officers/ Police/Army, teachers)<br>10 = others (Please specify):	
1.5 What is your ethnicity	1 = Manggarai 2 = Timor 3 = Sumba<br>4 = Bajawa 5 = Ende 6 = Ngada<br>7 = Alor 8 = Rote<br>9 = Other (Please specify):	
1.6 What is your monthly income (approximately)	Indonesian currency :	
1.7 What is your household monthly income (approximately)	Indonesian currency :	
1.8 What is the main material of the floor in your house?	1 = Ceramics<br>2 = Plastering cement<br>3 = Board<br>4 = Soil<br>5 = Others (Please specify) :	
1.9 What is the main material of the wall of your house?	1 = Plastering cement<br>2 = Zinc<br>3 = Bamboo /bamboo webbing/ rattan<br>4 = Wood/board/plywood<br>5 = Others (Please specify):	
1.10 What is the main material of the ceiling of your house?	1 = Concrete<br>2 = Wood/Plywood<br>3 = Bamboo webbing<br>4 = None<br>5 = Others (Please specify) :	
1.11 From the following household items, how many items do you have? Number = ………	1 = Radio 2 = TV 3 = TV cable<br>4 = Bike 5 = Motorbike<br>6 = Car 7 = Mobile phone.<br>8 = Electricity 9 = Fridge<br>10 = AC 11 = Tractor 12 = Generator	
1.12 What is the main source of drinking water for your family?	1 = Running tap water in dwelling<br>2 = Running tap water of public<br>3 = Drilled well pump<br>4 = Well<br>5 = Spring<br>6 = River<br>7 = Irrigation<br>8 = Other (Please specify)	
1.13 What is the nearest health service of your residence?	1 = Public Health centres<br>2 = Subsidiary Public Health centres	

	3 = Village health Post<br>4 = Midwife practices<br>5 = Village maternity posts<br>6 = Polyclinic<br>7 = General practice<br>8 = Other (Please specify):	
1.14 What is the distance to the nearest health facility?	……. (Distance in Km)	
1.15 What is your smoking status?	1 = Never smoke<br>2 = Current smoker (i.e., leaf or cigarette)<br>3 = Past smoker	
1.16 Do you currently suffering from any health condition?	1= Yes<br>2 = No	
1.17 If you answer yes on question 1.16, what is the current medical condition? (Please mention your disease):		

**SECTION 2: GENERAL KNOWLEDGE ABOUT MALARIA**		
2.1 Have you heard about malaria before?	1=Yes 2 = No	
If you said yes to question 2.1, please complete question 2.2 to 2.13. Otherwise go to section 3		
2.2 Where did you hear about malaria?	1 = TV<br>2 = Radio<br>3 = Newspaper<br>4 = Friend/Family member<br>5 = Local health facility<br>6 = School<br>7 = Church<br>8 = Mosque<br>9 = Community meetings<br>10 = Pamphlets<br>11 = Other (Please specify) :	
2.3 How would you rank the health effect of malaria?	1 = No effect<br>2 = Mild health effect (not so dangerous)<br>3 = Severe health effect (dangerous)	
2.4 Do you know some symptoms of malaria?	1 = Yes 2 = No	
2.5 If yes to question 2.4, can you please mention some symptoms of malaria? Tick the number of correct answers:	1 = 0<br>2 = 1<br>3 = >=2	
2.6 What do you think are the main symptoms of malaria infection?	1=Fever 2 = Shivering<br>3= Sweating 4 = Headache<br>5=Nausea and vomiting 6 = Diarrhoea<br>7=Dizziness 8 = Loss of appetite<br>9=Body weakness 10 = Joint pains<br>11 = Other (Please specify)	
2.7 In your opinion, what is the main causes of malaria?	1 = Mosquito bite<br>2 = Witchcraft<br>3 = Drinking dirty water<br>4 = Cold weather<br>5 = Unhygienic environment<br>6 = Close contact with a malaria sufferer<br>7 = Other(Please specify) :	
2.8 Do you think you have enough information on malaria?	1 = Yes 2 = No	
2.9 If you answer no to question 2.8, what information would you like to get?	1 = Information on transmission<br>2 = Information on prevention<br>3 = Information on control	

	4 = Information on treatment<br>5 = Information on signs and symptoms<br>6 = any information<br>7= other (Please specify):	
2.10 How would you like this information to be communicated to you? (Through what channels of communication?)	1 = TV<br>2 = Radio<br>3 = Newspaper<br>4 = Friend/Family member<br>5 = Local health facility<br>6 = School<br>7 = Church<br>8 = Mosque<br>9 = Community meetings<br>10 = Pamphlets<br>11 = Other (Please specify) :	
2.11 Do you think malaria can be prevented?	1 = Yes 2 = No	
2.12 If yes to question 2.11, can you please mention some protective measures to guard against malaria? Tick the number of protective measures mentioned:	1 = 0<br>2 = 1<br>3 = >=2	
2.13 What personal protective measures can you use to guard against malaria infection?	1 = Sleeping with non-insecticide treated net<br>2 = Sleeping with insecticide treated net received <=3 year ago<br>3 = Sleeping with insecticide treated net received > 3 year ago<br>4 = Using mosquito coil<br>5 = Keep house clean<br>6 = Covering ventilation with anti-mosquito nets<br>7 = Using Indoor Residual Spraying<br>8 = Wearing long sleeved clothes when go outdoors at night<br>9 = Taking anti-malaria drugs when staying at malaria endemic areas.<br>10 = Other (Please specify) :	

**SECTION 3: TREATMENT SEEKING BEHAVIOUR**		
3.1 If you or member of your family present with the signs and symptoms of malaria would you/they seek treatment?	1 = Yes<br>2 = No	
3.2 If yes to 3.1, how soon after suspecting that you or your family member are infected with malaria would you/they seek treatment?	1 = One day (Within 24 hours)<br>2 = 2 days<br>3 = 3 days<br>4 = 4 days or more<br>5 = I did not go for treatment	
3.3 If yes to 3.1 and you or member of your family present with the signs and symptoms of malaria where would you/they seek treatment?	1 = Public health facilities<br>2 = Private health facilities<br>3 = Traditional healer<br>4 = Pharmacy<br>5 = self-treat<br>6 = other (Please specify):	

**SECTION 4: PRACTICE OF PERSONAL PROTECTION**		
4.1 Does anyone in the household use any personal protective items to help prevent malaria infection?	1 = Yes 2 = No	
4.2 If yes on the question 4.1, can you please mention some protective measures to guard against malaria? Tick number of protective measures mentioned:	1 = 0<br>2 = 1<br>3 = >=2	
4.3 What personal protective measures do you use to guard against malaria infection?	1 = Sleeping with non-insecticide treated net<br>2 = Sleeping with insecticide treated net received <=3 year ago<br>3 = Sleeping with insecticide treated net received > 3 year ago<br>4 = Using mosquito coil/electric anti mosquito mats<br>5 = Keep house clean<br>6 = Covering ventilation with anti-mosquito nets<br>7 = Using Indoor Residual Spraying<br>8 = wearing long sleeved clothes when go outdoors at night<br>9 = Taking anti-malaria drugs when staying at malaria endemic areas.<br>10 = Other (Please specify) :	
4.4 Have your household received indoor residual spraying ever?	1 = Yes 2 = No	
4.5 If yes on question 4.4, when did it happen?	Month ……….. Year ……	
4.6 Do you have any mosquito net?	1 = Yes 2 = No	
4.7 What type of mosquito net do you have?	1 = Long Lasting Insecticide-Treated Bet Nets(LLINs)<br>2 = Non LLINs<br>3 = LLINs and Non LLINs	
**4.8 If you choose option 1 to question 4.7,**<br>**a. How many mosquito nets do you have?**<br>**b. When did you get this bed net:**<br>**c. Does this bed net eligible to be used?**<br>**d. How many sleeping groups do you have?**	 ……… (Number of LLINs)<br>Year ……<br>1 = Yes 2 = No<br>…………..(Number of sleeping groups)	
**4.9 If you choose option 2 to question 4.7,**<br>**a. How many mosquito nets do you have?**<br>**b. Does this bed net eligible to be used?**	 …………… (Number of non-LLINs)<br>1 = Yes 2 = No	
If you choose option 3 to question 4.7, please complete questions 4.8 and 4.9		
4.10 a. Did you sleep under bed net last night?<br>***b. If yes to question 4.10a, What type of bed net do you use?***	1 = Yes 2 = No<br>1= LLINs 2 = Non LLINs	
4.11 Did all of your family members sleep under mosquito net last night?	1 = Yes 2 = No	
4.12 If you said no to question 4.10a, what is the main reason?	1 = Too hot<br>2 = Bed net was not hung up<br>3 = Bed net was not in good condition /torn<br>4 = Mosquito net is for children<br>5 = Mosquito net is for pregnant women<br>6 = No mosquitoes<br>7 = Not get used to<br>8 = other (Please specify):	
4.13 Did you sleep indoors or outdoors last night?	1 = Indoors 2 = Outdoors	

4.14 To the nearest hour, what time did you go to sleep last night?	1 = 6-7 pm 2 = 7-8 pm 3 = 8-9 pm<br>4 = 9-10 pm 5 = 10-11pm<br>6 = 11 -12 mid night 7 = after 12 mid night	
4.15 To the nearest hour, what time did you wake up today?	1 = Before 4 am 2 = 4 – 5 am 3 = 5 – 6 am<br>4 = 6-7 am 5 = 7- 8 am 6 = after 8 am	
4.16 What time did you finally go indoors for the night?	1 = 6-7 pm 2 = 7-8 pm 3 = 8-9 pm<br>4 = 9-10 pm 5 = 10-11pm<br>6 = 11 -12 mid night 7 = after 12 mid night	
4.17 What time did you first go outdoors for the day this morning?	1 = Before 4 am 2 = 4 – 5 am 3 = 5 – 6 am<br>4 = 6-7 am 5 = 7- 8 am 6 = after 8 am	
4.18 What is your main activities before you go to sleep?	1 = Relaxing<br>2 = Storytelling<br>3 = Watching TV<br>4 = Preparing meals<br>5 = Eating<br>6 = Weaving<br>7 = Fetching water or firewood<br>8 = Other (Please specify) :	
4.19 Did you have travel history in the last one month?	1 = Yes 2 = No	

**SECTION 5 : PRACTICE FOR MALARIA TREATMENT**		
5.1 Have you ever suffered from malaria?	1 = Yes 2 = No	
If you answer yes on question 5.1, please do question 5.2 to 5.11, otherwise go to section 6		
5.2 When did it occur?	Month: …….. Year: …….	
5.3 What symptoms did you have then?	1 = Fever 2 = Shivering<br>3 = Sweating 4 = Headache<br>5 = Others (Please specify):	
5.4 How soon after suffering from malaria did you seek treatment?	1 = One day (Within 24 hours)<br>2 = 2 days<br>3 = 3 days<br>4= 4 days or more<br>5 = I did not go for treatment	
5.5 If you sought treatment, where did you go to seek treatment?	1 = Public health facilities<br>2 = Private health facilities<br>3 = Pharmacy<br>4 = Traditional healer<br>5 = self-treatment<br>6 = other (Please specify):	
5.6 If your response to 5.5 was option 1 or 2 or 3 , did you receive blood test?	1 = Yes 2 = No	
5.7 If your response to 5.4 was option 1 or 2 or 3, did you receive any medicine?	1 = Yes 2 = No	
5.8 If you responded yes to 5.6, did you receive the medicine stated below?<br>***5.8.1 Artemisinin-based Combination Treatment (ACT) + 1 day Primaquin***<br>***5.8.2 Artemisinin-based Combination Treatment (ACT) + 14 days Primaquin***<br>***5.8.3 Other medicine (Please specify):***	 <br>1 = Yes 2 = No<br><br>1 = Yes 2 = No	

5.9 Did you consume the medicine in question 5.7 until finish?	1 = Yes 2 = No	
5.10 If you answered no to question 5.8, what was the main reason for that?	1 = Forgot to take<br>2 = Felt better<br>3 = Not comfortable due to drug's side effects<br>4 = Other (Please specify) :	
5.11 If you choose option 4 to question 5.4, what is the main reason to do that?	1 = Tradition<br>2 = Desperation with modern medication<br>3 = No other health service available<br>4 = More potent<br>5 = The disease is not severe<br>6 = Safer<br>7 = Cheaper<br>8 = Trying<br>9 = Other (Please specify):	

**SECTION 6: INFORMATION ABOUT HOUSEHOLD MEMBERS**		
6.1 What is your family size?	The number of family members:	
6.2 How many children in your family under five years of age?	The number of children under 5 year :	
6.3 Does your household have at least one pregnant women?	1= Yes 2 = No	
6.4 Information on your spouse (Husband/Wife)		
6.4.1. What is his/her age (in years):		
6.4.2. What is his/her Sex?	1 = Male 2 = Female	
6.4.3. What is his/her highest level of education?	1 = No education 4 = Senior High School<br>2 = Primary school 6 = Diploma<br>3 = Junior High school 6 = Bachelor or above	
6.4.4. What is his/her main occupation?	1= Farmer 2 = housewife<br>3= Fisherman 4 = Labour<br>6 = Trader/Merchandiser<br>6 = Student 7 = Unemployed<br>8 = unable to work<br>9 = Govt. or non-govt. employment (Officers/ Police/Army, teachers)<br>10 = others (Please specify):	
6.4.5. What is his/her smoking status?	1 = Never smoke<br>2 = Current smoker (i.e., leaf or cigarette)<br>3 = Past smoker	
6.4.6 Does she/he currently suffering from any health condition?	1= Yes 2 = No	
6.4.7 If you answer yes on question 6.4.6., what is the current her/his medical condition? (Please mention her/his disease):		
6.4.8 Has she/he suffered from malaria ever?	1= Yes 2 = No	
6.4.9 If you answer yes on question 6.4.8, when it did it occur?	Year	
6.4.10 Does she/he sleep under bed net last night?	1= Yes 2 = No	
6.4.11 What type of bed net does she/he use?	1= LLINs 2 = Non LLINs	
6.4.12 Did he/she had travel history in the last one month?	1= Yes 2 = No	

Child # 1

6.5 Information on your child under age of five		
c1 6.5.1. What is his/her age (in years):		
c1 6.5.2. What is his/her Sex?	1 = Male 2 = Female	
c1 6.5.3 Does she/he currently suffering from any health condition?	1= Yes 2 = No	
c1 6.5.4 If you answer yes on question c1 6.5.3., what is the current her/his medical condition? (Please mention her/his disease):		
c1 6.5.5 Has she/he suffered from malaria?	1= Yes 2 = No	
c1 6.5.6 If you answer yes on question c1 6.5.5, when it did it occur?		
c1 6.5.7 Did she/he sleep under bed net last night?	1= Yes 2 = No	
c1 6.5.8 What type of bed net does she/he use?	1= LLINs 2 = Non LLINs	

Child # 2

c2 6.5.1. What is his/her age (in years):		
c2 6.5.2. What is his/her Sex?	1 = Male 2 = Female	
c2 6.5.3 Does she/he currently suffering from any health condition?	1= Yes 2 = No	
c2 6.5.4 If you answer yes on question c2 6.5.3., what is the current her/his medical condition? (Please mention her/his disease):		
c2 6.5.5 Has she/he suffered from malaria?	1= Yes 2 = No	
c2 6.5.6 If you answer yes on question c2 6.5.5, when it did it occur?		
c2 6.5.7 Did she/he sleep under bed net last night?	1= Yes 2 = No	
c2 6.5.8 What type of bed net does she/he use?	1= LLINs 2 = Non LLINs	

Child # 3

c3 6.5.1. What is his/her age (in years):		
c3 6.5.2. What is his/her Sex?	1 = Male 2 = Female	
c3 6.5.3 Does she/he currently suffering from any health condition?	1= Yes 2 = No	
c3 6.5.4 If you answer yes on question c3 6.5.3., what is the current her/his medical condition? (Please mention her/his disease):		
c3 6.5.5 Has she/he suffered from malaria?	1= Yes 2 = No	
c3 6.5.6 If you answer yes on question c3 6.5.5, when it did it occur?		
c3 6.5.7 Did she/he sleep under bed net last night?	1= Yes 2 = No	
c3 6.5.8 What type of bed net does she/he use?	1= LLINs 2 = Non LLINs	

Note: Keep cell empty if there is no child and add information if there are more than three children

THANK YOU VERY MUCH

We have to come to the end of our interview. Your time, honest opinions and valuable contributions are highly appreciated. Do you have any questions?
